# Ultrathin, High‐Aspect‐Ratio Bismuth Sulfohalide Nanowire Bundles for Solution‐Processed Flexible Photodetectors

**DOI:** 10.1002/advs.202403463

**Published:** 2024-07-04

**Authors:** Da Won Lee, Seongkeun Oh, Dong Hyun David Lee, Ho Young Woo, Junhyuk Ahn, Seung Hyeon Kim, Byung Ku Jung, Yoonjoo Choi, Dagam Kim, Mi Yeon Yu, Chun Gwon Park, Hongseok Yun, Tae‐Hyung Kim, Myung Joon Han, Soong Ju Oh, Taejong Paik

**Affiliations:** ^1^ Department of Intelligent Semiconductor Engineering Chung‐Ang University Seoul 06974 Republic of Korea; ^2^ Department of Materials Science and Engineering Korea University Seoul 02841 Republic of Korea; ^3^ Department of Physics Korea Advanced Institute of Science and Technology (KAIST) Daejeon 34141 Republic of Korea; ^4^ School of Integrative Engineering Chung‐Ang University Seoul 06974 Republic of Korea; ^5^ Department of Biomedical Engineering SKKU Institute for Convergence Sungkyunkwan University (SKKU) Suwon Gyeonggi 16419 Republic of Korea; ^6^ Department of Intelligent Precision Healthcare Convergence SKKU Institute for Convergence Sungkyunkwan University (SKKU) Suwon Gyeonggi 16419 Republic of Korea; ^7^ Department of Chemistry and Research Institute for Convergence of Basic Science Hanyang University Seoul 04763 Republic of Korea

**Keywords:** flexible devices, nanobundles, nanocrystal inks, nanowires, semiconductors

## Abstract

In this study, a novel synthesis of ultrathin, highly uniform colloidal bismuth sulfohalide (BiSX where X = Cl, Br, I) nanowires (NWs) and NW bundles (NBs) for room‐temperature and solution‐processed flexible photodetectors are presented. High‐aspect‐ratio bismuth sulfobromide (BiSBr) NWs are synthesized via a heat‐up method using bismuth bromide and elemental S as precursors and 1‐dodecanethiol as a solvent. Bundling of the BiSBr NWs occurs upon the addition of 1‐octadecene as a co‐solvent. The morphologies of the BiSBr NBs are easily tailored from sheaf‐like structures to spherulite nanostructures by changing the solvent ratio. The optical bandgaps are modulated from 1.91 (BiSCl) and 1.88 eV (BiSBr) to 1.53 eV (BiSI) by changing the halide compositions. The optical bandgap of the ultrathin BiSBr NWs and NBs exhibits blueshift, whose origin is investigated through density functional theory‐based first‐principles calculations. Visible‐light photodetectors are fabricated using BiSBr NWs and NBs via solution‐based deposition followed by solid‐state ligand exchanges. High photo‐responsivities and external quantum efficiencies (EQE) are obtained for BiSBr NW and NB films even under strain, which offer a unique opportunity for the application of the novel BiSX NWs and NBs in flexible and environmentally friendly optoelectronic devices.

## Graphical abstract

The colloidal synthesis of ultrathin high‐aspect‐ratio bismuth sulfohalide (BiSX, where X = Cl, Br, and I) nanowires (NWs) and NW bundles (NBs) are represented for the first time. The morphologies and optical properties of the BiSX NWs and NBs are easily tailored by changing the reaction parameters and compositions. Toxic‐metal‐free highly efficient flexible photodetectors are fabricated using room‐temperature solution processes.

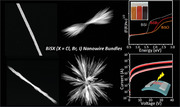

## 1 Introduction

Semiconducting inorganic nanowires (NWs) have gained significant attention as attractive building blocks for the fabrication of electronic and optoelectronic devices via bottom‐up integration due to their unique electronic, optical, and mechanical properties.^[^ ^1^, ^2^, ^3^ ^]^ The high aspect ratios of anisotropic 1D structures enable efficient charge transport and electrical conductivity, rendering these structures ideal building blocks for transistors,^[^ ^4^ ^]^ memory devices,^[^ ^5^ ^]^ and logic gates.^[^ ^6^ ^]^ Furthermore, colloidal semiconducting NWs exhibit exceptional optical properties including quantum confinement effects and strong light‐matter interactions, which render these NWs promising candidates for light‐emitting diodes,^[^ ^7^ ^]^ photovoltaics,^[^ ^8^ ^]^ photodetectors,^[^ ^9^ ^]^ and nanolasers.^[^ ^10^ ^]^ The ordered arrays or bundles formed by assembling colloidal semiconducting NWs demonstrate high electrical conductivities or electrochemical properties as compared to those of individual NWs owing to the interconnectivity between NWs.^[^ ^11^, ^12^, ^13^, ^14^ ^]^ Additionally, because of their hierarchical natures, NW arrays and bundles demonstrate mechanical flexibilities, enabling their use in flexible and stretchable electronic devices with superior electronic performances.^[^ ^15^ ^]^

Thus far, various synthetic approaches have been reported for semiconducting NWs such as template‐assisted growth^[^ ^16^ ^]^ and vapor‐liquid‐solid growth method.^[^ ^17^ ^]^ Among them, colloidal syntheses exhibit substantial potential for low‐temperature, scalable fabrication of semiconducting nanocrystals (NCs) with tailored dimensions, compositions, and crystal structures,^[^ ^18^, ^19^, ^20^, ^21^, ^22^ ^]^ which facilitate precise control of their electronic and optical properties. Moreover, colloidal NWs provide a unique opportunity for the relatively low‐temperature, inexpensive, and large‐area solution‐processed fabrication of functional devices on flexible polymeric substrates or textiles for applications in wearable electronic devices.^[^ ^23^ ^]^ To date, a wide range of colloidal semiconducting NWs have been synthesized, for instance, chalcogenide^[^ ^24^, ^25^, ^26^ ^]^ and perovskite NWs^[^ ^27^, ^28^ ^]^ with excellent electronic and optoelectronic properties. However, many of these materials still suffer from critical limitations, for example, the use of toxic heavy metals, including lead (Pb) and cadmium (Cd), which has raised significant environmental and health concerns, and lack of colloidal stability, limiting the large‐scale integration of colloidal NWs into functional devices. Therefore, efficient and scalable synthetic methods for colloidal NWs with non‐toxic elements and high electronic performances.

Recently, pnictogen chalcohalides (V–VI‐VII where V = Bi, Sb; VI = S, Se, Te; and VII = Cl, Br, I) have attracted considerable attention as novel semiconducting colloidal NCs owing to their toxic heavy‐metal‐free compositions, facile tunings of crystal and electronic structures over a wide range via adjustment of the three components, and high stabilities.^[^ ^29^, ^30^ ^]^ Pnictogen chalcohalides possess high‐Z ns^2^ cations with d^10^s^2^p^0^ electronic configuration and high levels of band dispersion originating from hybridization among the anion or cation‐p states.^[^ ^31^ ^]^ Consequently, pnictogen chalcohalides exhibit strong absorption coefficients, high defect tolerances, and high carrier transport properties, facilitating their application in optoelectronic devices.^[^ ^32^, ^33^ ^]^ Specifically, bismuth (Bi) is considered to be a non‐toxic heavy metal and extensively used in cosmetics, solders, pigments, catalysts, devices, and medicines.^[^ ^34^, ^35^ ^]^ Solution‐phase colloidal syntheses of bismuth chalcohalide (BiSBr) nanorods via hot‐injection of chalcogen and halogen precursors into Bi‐carboxylate complexes were reported by Giansante et al.,^[^ ^36^ ^]^ and to date, many researchers have employed these approaches.^[^ ^37^, ^38^, ^39^ ^]^ Nevertheless, the electronic and optoelectronic properties of colloidal bismuth chalcohalides with different morphologies and compositions have rarely been investigated. Additionally, very limited studies have been reported on the syntheses of highly flexible, colloidally dispersed, high‐aspect‐ratio bismuth chalcohalide NWs and integrations of these NWs into functional optoelectronic devices fabricated via room‐temperature solution processing.

Herein, we report the colloidal synthesis of bismuth sulfohalide (BiSX; X = Cl, Br, I) NWs and NW bundles (NBs) via an injection‐free heat‐up process. First, the reaction was performed using bismuth bromide (BiBr_3_) and sulfur (S) as precursors in a solvent mixture of 1‐dodecanethiol (DT) and 1‐octadecene (ODE). Our heat‐up synthesis of colloidal BiSBr NWs and NBs requires relatively lower temperatures (typically <150 °C) and very short reaction times (<10 min), demonstrating significant commercialization potential for mass production. The relative ODE:DT volume ratio in the solvent mixture played an important role in the formation of high‐aspect‐ratio ultrathin BiSBr NWs and NBs. By changing the relative ODE:DT volume ratio, the morphologies of separated BiSBr NWs were converted into sheaf‐like BiSBr NBs and then to spherulite BiSBr NBs. Furthermore, 1D high‐aspect‐ratio BiSCl and BiSI NWs were synthesized by varying the precursors, tuning the bandgaps of BiSX NWs from visible to near‐infrared. The ultrathin BiSBr NWs showed a blue shift in the optical bandgap, and the reason for this blue shift was further investigated using first‐principles calculations. The as‐synthesized BiSBr NWs and NBs exhibited high colloidal stabilities in nonpolar solvents. We demonstrated the room‐temperature solution‐processed fabrication of flexible photodetectors using colloidal BiSBr NWs and NBs and analyzed the optoelectronic properties of these photodetectors. Via electric field alignments (EFAs) of 1D BiSBr NWs and NBs followed by solid‐state ligand exchange, BiSBr NWs and NBs with high responsivities and external quantum efficiencies (EQEs) were achieved, and their high photoresponsivities were maintained even under significant strains on flexible substrates. These results indicate promising potentials of our bismuth sulfohalide NWs and NBs for application in many emerging optoelectronic devices and flexible electronics.

## 2 Results

### 2.1 Synthesis of Ultrathin and Highly Uniform BiSBr NWs and NBs

Colloidal BiSBr NWs were synthesized via a heat‐up process using DT as a solvent.^[^ ^39^ ^]^ Before the reaction, the mixtures of precursors and solvents were pre‐heated at 80 °C under vacuum to remove volatile solvents and water. After degassing, the reaction solution was heated up to 120 °C under a nitrogen atmosphere and maintained at this temperature for 3 min for the reaction. During heating, the reaction solution turned from transparent to reddish, indicating the formation of BiSBr NWs. BiSBr NWs were purified by adding toluene followed by centrifugation at 8000 rpm for 3 min to obtain phase‐pure BiSBr NWs. DT solvent has been frequently used as a S source for the synthesis of semiconducting metal chalcogenide NCs such as CdS,^[^ ^40^ ^]^ Cu_2_S,^[^ ^41^ ^]^ Ag_2_S,^[^ ^42^ ^]^ and CuInS.^[^ ^43^ ^]^ However, BiSBr NWs were not formed in the absence of S precursors under our experimental conditions. Generally, sulfidation of metal chalcogenide NCs using DT proceeds at temperatures higher than 200 °C. As our reaction was conducted at temperatures below this temperature range, S precursors were added to the reaction mixture to achieve sulfohalide NWs.

**Figure 1:**
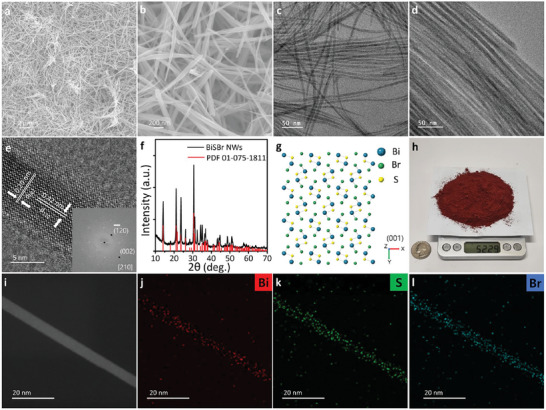
a,b) SEM and c,d) TEM images of BiSBr NWs. e) HRTEM image of BiSBr NWs along the [210] zone axis. The inset represents the FFT of the HRTEM image. f) XRD pattern of the BiSBr NWs. g) Atomistic model of orthorhombic BiSBr. h) Large‐scale synthesis of BiSBr NWs to obtain 52.29 g in a single reaction batch. The reaction was performed at 120 °C for 3 min. i) STEM image of a single BiSBr NW and EDS elemental mapping images of j) Bi, k) S, and l) Br in a single BiSBr NW.

**Figure** **1**a–d shows the scanning electron microscopy (SEM) and transmission electron microscopy (TEM) images of BiSBr NWs synthesized using DT. Ultrathin BiSBr NWs demonstrated excellent flexibilities and uniform widths and lengths. The average widths and lengths of BiSBr NWs, which were determined by statistical analysis of TEM images, were 5.2 ± 0.9 nm and 3.89 ± 0.79 µm, respectively (Figure S1, Supporting Information). High‐resolution TEM (HRTEM) image of a single BiSBr NW revealed a single‐crystalline structure with the orthorhombic crystal space group Pnam #62 and interplanar distances of 0.20 and 0.42 nm for (002) and (120) planes, respectively (Figure 1e and inset image). Fast Fourier transform (FFT) of the HRTEM image appropriately matched the simulated electron diffraction (ED) pattern with a [210] zone axis (Figure S2, Supporting Information). The FFT results indicated anisotropic growths of BiSBr NWs along the c‐axis.^[^ ^29^ ^]^ X‐ray diffraction (XRD) pattern of BiSBr NWs also matched that of orthorhombic BiSBr (PDF 01‐075‐1811; Figure 1f,g). The diffraction peaks at 14, 21, 23, and 30° were assigned to the (110), (120), (210), and (121) planes of BiSBr NWs, respectively, in the absence of impurities. Relatively low temperatures, short reaction times, and a simple injection‐free process enable the large‐scale synthesis of conducting NWs. We synthesized 52.29 g of BiSBr NWs in a single batch with 120 °C and 3 min as reaction conditions, demonstrating their considerable potential for mass production. (Figure 1h) Scanning TEM (STEM)‐energy‐dispersive X‐ray spectroscopy (EDS) mappings revealed homogeneous distributions of Bi, S, and Br in a single NW, and elemental stoichiometry was 34.28%:32.02%:33.70%, representing a compositional ratio of 1:1:1 (Figure 1i–l).

**Figure 2:**
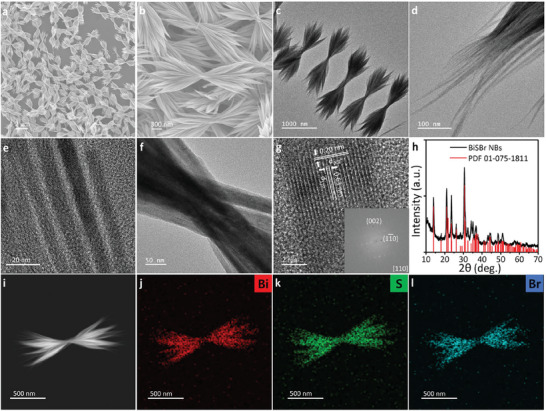
a,b) SEM and c–f) TEM images of BiSBr NBs. g) HRTEM image of a single NW view along [110] zone axis. Inset represents FFT of HRTEM image. h) XRD pattern of the BiSBr NWs. i) Dark‐field STEM and EDS mapping images of j) Bi, k) S, and l) Br.

Bundling of ultrathin, flexible BiSBr NWs was achieved using a solvent mixture of DT and ODE. **Figure** **2**a–d depicts the SEM and TEM images of the BiSBr NBs synthesized using 10 vol% DT and 90 vol% ODE. A dozen BiSBr NWs were bundled together in the middle to form sheaf‐like nanostructures. High‐magnification TEM image (Figure 2e,f) revealed that a single BiSBr NB comprised ultrathin flexible NWs, and multiple NWs were twisted at the center. HRTEM image of a single NW in a BiSBr NB also exhibited a single‐crystalline structure (Figure 2g). FFT of the HRTEM image indicated that the individual NWs in the bundle demonstrated the same crystal structure and growth direction as those of BiSBr NWs fabricated using DT. Furthermore, XRD results implied the existence of an orthorhombic BiSBr phase (PDF 01‐075‐1811, Figure 2h). Selected area electron diffraction (SAED) of BiSBr NBs was conducted at the tied region and the end of a bundle (Figure S3, Supporting Information). Multiple sets of ED spots were observed in the SAED pattern of the bundle edge due to the presence of multiple single‐crystalline NWs. Multiple sets of single‐crystalline ED spots were also noticed in the central region, which indicated the bundling of highly oriented BiSBr NWs in a single BiSBr NB. Figure 2i–l shows the dark‐field STEM image of a bundled BiSBr NW and elemental mapping images for Bi, S, and Br. Homogeneous distributions of Bi, S, and Br were observed in a single NB with stoichiometries of 33.67, 34.23, and 34.23%, respectively. Figure S4a–d (Supporting Information) depicts full survey and high‐resolution X‐ray photoelectron (XPS) results for Bi, S, and Br, respectively. Bi 4f (Bi 4f_5/2_ and Bi 4f_7/2_), S 2p (S 2p_3/2_ and S 2p_1/2_), and Br 3d (Br 3d_3/2_ and Br 3d_5/2_) peaks were noticed at the binding energies of 164.27 and 158.98 eV, 162.5 and 161.3 eV, and 69.1 and 68.1 eV, respectively. According to the XPS results, atomic percentages of Bi, S, and Br were 34.2, 32.04, and 33.75%, respectively, which were consistent with the stoichiometries of BiSBr NWs. These results indicated that the use of the mixed solvent (DT and ODE) led to the bundling of BiSBr NWs without significant changes in the crystal structures and stoichiometries of orthorhombic BiSBr NWs.

When the reaction was performed in 100 vol% ODE, BiSBr nanorods (NRs) with smaller aspect ratios and larger widths were acquired (Figure S5, Supporting Information). Average lengths and widths of BiSBr NRs were 987.3 ± 228.1 and 111.6 ± 31.5 nm, respectively, with broad size distributions. These results implied that all NWs or NRs were physically separated in the absence of bundling between NWs or NRs when ODE or DT was used alone, and the co‐presence of ODE and DT in the reaction mixture was a key factor for the bundling of BiSBr NWs. The presence of DT not only induced bundling, but also considerably reduced the widths of highly uniform BiSBr NWs, providing flexibility to these NWs. This might be attributed to the fact that DT acted as not only a reaction solvent but also a surfactant that coordinated the surfaces of BiSBr NWs, which induced preferential growth of BiSBr in the unidirectional direction along the c‐axis while preventing growth in other crystallographic directions. BiSBr NWs and NBs formed stable colloidal dispersion in nonpolar solvents, such as hexane, toluene, and chloroform, without precipitation, even during long‐term storage. Fourier transform infrared (FTIR) spectra of the as‐synthesized BiSBr NWs and NBs confirmed the presence of characteristic C‐H vibrations of the alkyl group, revealing the existence of DT ligands on the surfaces of these NWs and NBs (Figure S6, Supporting Information). This might indicate that the DT ligands on the large surface areas of ultrathin NBs endowed BiSBr NBs with excellent colloidal stabilities in nonpolar solvents.

### 2.2 Tailoring the Halide Compositions of BiSX NWs

**Figure 3:**
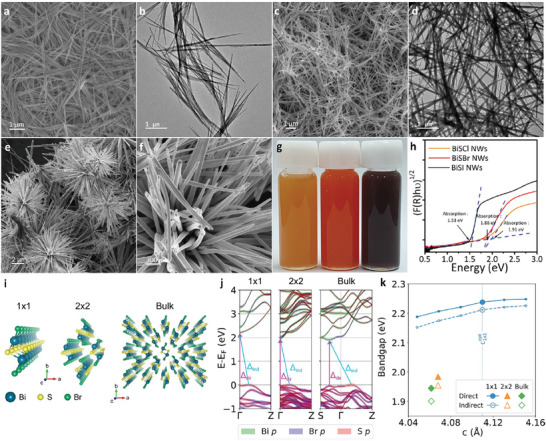
a) SEM and b) TEM images of BiSCl NWs. c) SEM and d) TEM images of BiSI NWs. e,f) SEM images of spherulite BiSI NBs. g) Photograph of colloidal solutions of BiSCl (orange), BiSBr (red), and BiSI (dark brown) NWs dispersed in toluene. h) Kubelka–Munk plot of BiSCl, BiSBr, and BiSI NWs. i) The crystal structure and j) the calculated band dispersion of 1 × 1 chain (left), 2 × 2 chain (middle), and bulk (right) BiSBr. The dark cyan, yellow, and green spheres in i) represent Bi, S, and Br atoms, respectively. The magenta and cyan arrows in j) represent the direct and indirect transitions, respectively. k) The calculated band gap at the optimized lattice parameters. The direct (indirect) band gap at the optimized structure of the 1 × 1 chain, 2 × 2 chain, and bulk BiSBr are denoted by the large filled (empty) markers of the blue circle, orange triangle, and green diamond, respectively. The optimized c lattice parameter (along the chain direction) is 4.110 Å, 4.068 Å, and 4.062 Å for the 1 × 1 chain, 2 × 2 chain, and bulk BiSBr, respectively. For 1 × 1 BiSBr, the evolution of direct (blue solid line) and indirect (blue dashed line) band gaps was also evaluated as a function of c considering the uniaxial strain ε from −1.5% (compressive) to 1% (tensile). The optimized lattice parameter c for 1 × 1 BiSBr ($c_{1\;\times \;1}^{\mathrm{opt}}$) is denoted by the blue vertical dotted line.

Halide compositions of bismuth chalcohalide NWs were further tailored by changing the reaction precursors. **Figure** **3**a–d shows the SEM and TEM images of BiSCl and BiSI NWs, respectively, synthesized using bismuth (III) chloride (BiCl_3_) and bismuth (III) iodide (BiI_3_) precursors in the presence of DT and ODE. Although ODE was used as a co‐solvent, BiSCl and BiSI were formed as NWs instead of NBs. SEM images indicated flexible NWs with average lengths and widths of 3.40 ± 0.64 µm and 17.4 ± 3.6 nm for BiSCl NWs and 4.41 ± 1.05 µm and 22.6 ± 5.4 nm for BiSI NWs, respectively. XRD pattern revealed a polymorphic structure of BiSCl^[^ ^36^ ^]^ and orthorhombic BiSI with a Pnam space group (JCPDS# 01‐073‐1711, Figure S7, Supporting Information). For the synthesis of high aspect‐ratio BiSI NWs, oleic acid (OA) was introduced into the solvent mixture of ODE and DT. As the bismuth(III) iodide (BiI_3_) precursors were not fully soluble in the ODE and DT solvent mixture, the reaction was conducted at temperatures above 250 °C, resulting in thick and rigid BiSI NRs. In contrast, the addition of OA facilitated the complete dissolution of BiI_3_ precursors in the solvent mixture, significantly reducing the BiSI NW formation temperature. When the reaction temperature was increased to 190 °C, spherulite BiSI NBs with thicker widths were generated (Figure 3e,f).

### 2.3 Optical Property Analysis and Theoretical Calculations

The optical bandgaps of BiSX NWs varied depending on the halide composition. Figure 3g displays the apparent colors of colloidal dispersion of BiSCl, BiSBr, and BiSI NWs in hexane solution. The diffuse reflectance spectra show that the absorption band edge was red‐shifted for heavier halide compositions among Cl, Br, and I. The bandgaps of BiSCl, BiSBr, and BiSI were 1.91 eV (649 nm), 1.88 eV (659 nm), and 1.53 eV (810 nm), respectively. These results reveal the versatile compositional diversities of BiSX NWs and NBs, which make it possible to tailor their optical and electronic properties for many emerging applications. No photoluminescence was observed from the BiSX NWs and NBs even at low temperatures (80 K), indicating that these materials featured indirect bandgaps. Interestingly, the optical bandgap of ultrathin BiSBr was blue‐shifted compared to that of thick NRs, from 1.97 to 2.05 eV for direct transition and from 1.79 to 1.88 eV for indirect transition (Figure S8, Supporting Information). This trend was only observed in ultrathin BiSBr NWs or NBs, and the optical bandgaps in BiSCl and BiSI NWs were identical to those of NRs, which implies that there is a relation between size and the bandgap of BiSBr. To understand these trends, we performed first‐principles calculations based on density functional theory (DFT). The thickness dependence of BiSBr NWs is modeled using the 1 × 1 chain, 2 × 2 chain, and bulk BiSBr, as shown in Figure 3i. The evolution of the band structure with increasing dimensionality is displayed in Figure 3j, where the different orbital characters are depicted with different colors. The valence band is mainly composed of S‐p and Br‐p, while the conduction band is composed of Bi‐p. This basic feature does not change significantly with dimensionality. Meanwhile, on moving from the 1 × 1 chain to the 2 × 2 chain, and to the bulk, the band gap size as well as the detailed dispersion are changed. In the cases of the chains, the valence band maxima (VBM) and the conduction band minimum (CBM) are found at the Γ‐Z and Γ points, respectively. In contrast, the bulk CBM is located in the S‐Γ line at a point shifted from Γ (see the right panel of Figure 3j). This systematic change in electronic structure is due to the reduced dispersion of the bottom‐most conduction and top‐most valence band as a function of dimensionality. Figure 3k shows that the direct band gap is gradually reduced as the dimension increases; Δ_dir_ = 2.24, 1.98, and 1.95 eV for 1 × 1, 2 × 2, and bulk BiSBr, respectively. The indirect band gap is also gradually reduced; Δ_ind_ = 2.21, 1.95, and 1.90 eV for 1 × 1, 2 × 2, and bulk, respectively. It should be noted that the indirect gap is always smaller than the direct gap for any dimensionality, which is consistent with the results of our experiment.

Moreover, Figure 3k clearly shows that, together with the band gap, the optimized c‐lattice parameter also gradually increases with a reduction in dimensionality. XRD measurements also indicated an increase in c‐lattice parameters in BiSBr NWs or NBs. Figure S9, (Supporting Information) shows a comparison of the XRD peak positions between ultrathin BiSBr NWs and thick BiSBr NRs. The peak positions of thin NWs containing c‐axis components were shifted to smaller angles compared to those of thick NRs, indicating the expansion of c‐lattice parameters in ultrathin BiSBr NWs. To investigate the structural dependence, we computed the direct and indirect band gaps by varying the c‐lattice parameter for 1 × 1 chain over the range of −1.5% to 1%, covering the optimized values of 2 × 2 and the bulk c‐lattice. The results are indicated by line‐connected circles in Figure 3k. While increasing the c‐lattice parameter slightly enlarges both direct (from 2.19 eV at −1.5% to 2.25 eV at 1% strain) and indirect gap (from 2.15 eV at −1.5% to 2.23 eV at 1% strain), its effect is not significant. However, when comparing the calculated band gaps of the 1 × 1 chain using the 2 × 2‐ and bulk‐optimized c‐lattice values with those of the 2 × 2 chain and bulk, notable enhancements were observed. Our result indicates that the enlarged band gap in the chain cannot be solely attributed to the strain effect; rather, it can be ascribed to bandwidth and dimensionality reduction, which can be also regarded as a quantum confinement effect. The underlying physical mechanism can be analyzed using the tight‐binding model parameters;^[^ ^44^, ^45^ ^]^ the limited interchain “hopping” and hybridization in lower dimensions effectively reduce the widths of both the conduction band (CB) and valence band (VB). Additionally, it results in a smaller energy splitting between the CB and VB, namely, the band gap. This behavior is distinctly visualized in our DFT calculation results depicted in Figure S10 (Supporting Information) and is in good agreement with the experiment result. It can also be interpreted as a quantum confinement effect, whose underlying physics is almost identical within the quantum well picture.^[^ ^46^ ^]^ In addition, the estimated exciton Bohr radius of bulk BiSBr is ≈1.5 nm (see computational details), which is slightly smaller than, but comparable with, the width of the BiSBr NW (≈5 nm), thus evidencing the quantum confinement effect. These results also provide a plausible explanation for the experimentally observed blueshift in the band gap of the thin BiSBr NWs relative to that of the thick BiSBr samples. Furthermore, our calculations predict that all BiSX (X = Cl, Br, I) samples exhibit the same trend of enlarged band gap with decreasing thicknesses, as shown in Table S1 (Supporting Information), although successful size control for X = l and CI could not be achieved experimentally.

### 2.4 Controlling the Shape of BiSBr NBs

**Figure 4:**
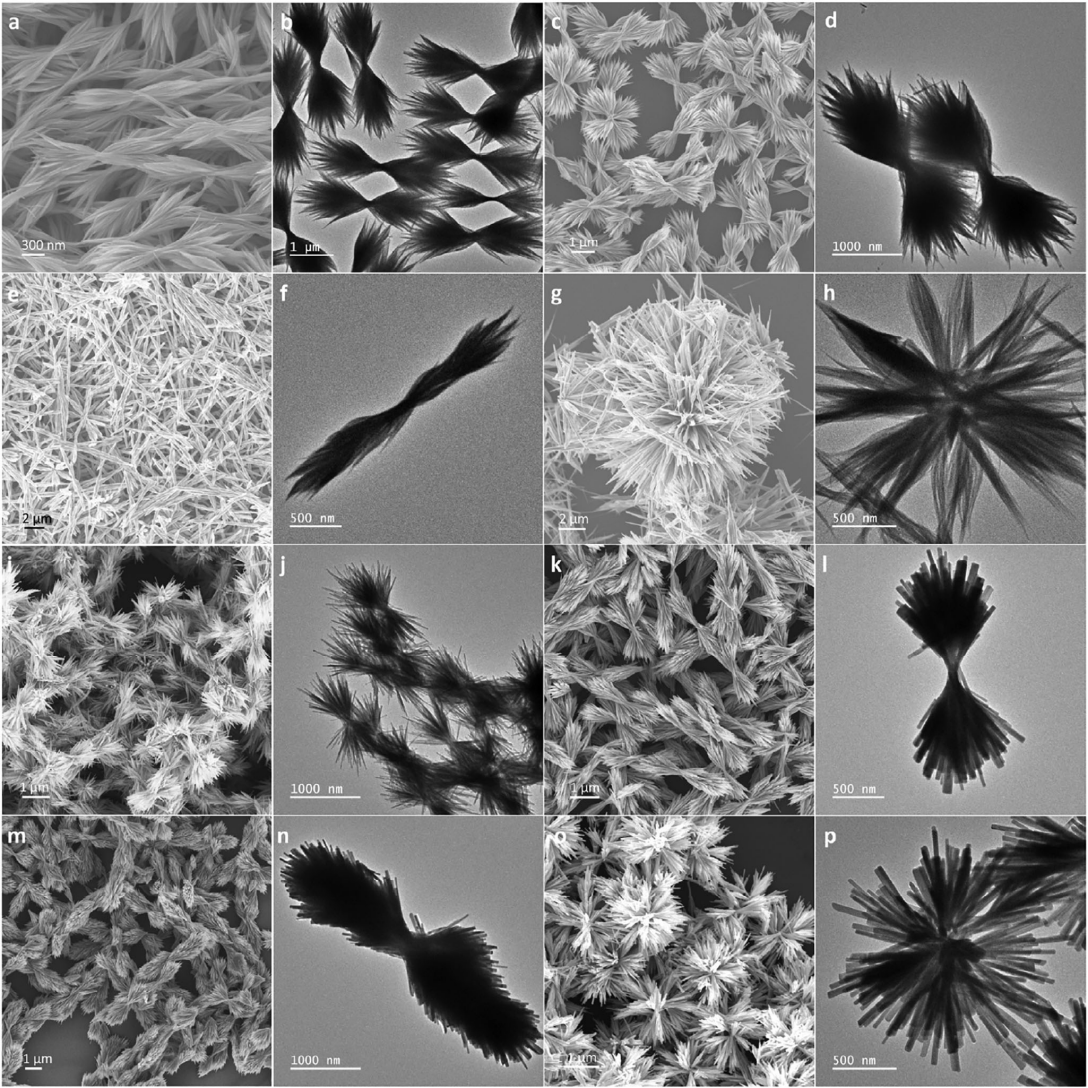
SEM and TEM images of BiSBr NBs synthesized at 120 °C using ODE:DT volume ratios of a and b) 90:10, c and d) 70:30, e and f) 40:60, and g and h) 10:90, and BiSBr NBs synthesized at 150 °C using ODE:DT volume ratios of i and j) 90:10, k and l) 70:30, m and n) 40:60, and o and p) 10:90. All reactions were performed for 3 min.

To understand the growth behaviors of BiSBr NBs, we systematically changed the ODE:DT volume ratio in the reaction mixture and characterized the morphologies of the resulting BiSBr NBs. **Figure** **4** shows the SEM and TEM images of the BiSBr NBs synthesized using different ODE:DT volume ratios. As described earlier, when an ODE:DT ratio of 90:10 was employed, highly uniform BiSBr NBs with average tip‐to‐tip distances of 3.09 ± 0.33 µm were achieved (Figure 4a,b). The average widths of individual BiSBr NWs were ≈6.9 ± 1.5 nm, which were similar to those of the BiSBr NWs prepared using DT alone. The widths of the tied regions of the bundles were ≈176.2 ± 28.6 nm, and all BiSBr NWs were tied in the center with approximately equal lengths on the left and right sides of the bundles. When the relative concentration of DT was increased to 30 vol%, the number of BiSBr NWs in an NB increased (Figure 4c,d). Widths of the tied NWs in the middle of NBs increased to ≈233.7 ± 34.1 nm. Tip‐to‐tip distances of BiSBr NBs were ≈3.14 ± 0.19 µm, which were similar to those of the BiSBr NBs constructed using 10% DT in the solvent mixture. When the relative concentration of DT was further increased to 60 vol%, bundled parts of the NWs appeared at several locations in addition to the centers of NWs (Figure 4e,f). The tip‐to‐tip distances were ≈3.86 ± 0.44 µm with central widths of 261.0 ± 0.52 nm. When 90 vol% DT was used, multiple BiSBr NBs were bundled together to form spherulite structures (Figure 4g,h).

When the reaction temperature was increased to 150 °C, bundling of BiSBr NWs was still observed. Figure 4i–p shows the SEM and TEM images of the BiSBr NBs synthesized at 150 °C using different ODE:DT volume ratios. BiSBr NWs bundled at the centers to form sheaf‐like or spherulite structures. Widths of the tied regions of NBs were ≈158.5 ± 29.9, 244.7 ± 34.6, and 305.6 ± 45.3 nm when 10, 30, and 60 vol% DT was employed, respectively. When 90 vol% DT was used, NWs produced spherulite structures, and consequently, the widths of the centers could not be accurately measured. However, the trend where a higher volume fraction of DT resulted in an increase in the number of NWs in an NB was consistently observed. As noticed in the TEM images, BiSBr NBs consisted of thicker NWs. Average widths of BiSBr NWs were ≈19.3 ± 4.7, 38.2 ± 9.2, 40.1 ± 9.8, and 40.5 ± 6.3 nm when 10, 30, 60, and 90 vol% DT was used, respectively. This may be attributed to the increased growth rates along the lateral direction at higher reaction temperatures. Nevertheless, the average tip‐to‐tip distances of BiSBr NBs did not significantly vary with respect to the reaction temperature. These results revealed that the lateral growth rates of BiSBr NWs were more temperature‐sensitive than the growth rates along the c‐axis under our reaction conditions. Although individual BiSBr NWs were stiff and straight, all NWs were still tied at the centers, forming sheaf‐like bundles. In addition, the optical bandgap of these BiSBr NBs were identical to those of BiSBr NRs and no shift of diffraction peak positions were observed, indicating that the expansion c‐lattices and the blue shift of optical bandgap were only observed in ultrathin NWs.

### 2.5 Plausible Formation Mechanism of BiSBr NBs

Bundling of chalcogenide NWs has been reported for bismuth sulfide (Bi_2_S_3_),^[^ ^47^ ^]^ antimony sulfide and selenide (Sb_2_S_3_ and Sb_2_Se_3_),^[^ ^48^, ^49^, ^50^ ^]^ and tellurium (Te) nanostructures.^[^ ^51^, ^52^ ^]^ Tang. et al. reported the formation of sheaf‐like Bi_2_S_3_ nanostructures via the crystal splitting during growth.^[^ ^53^ ^]^ These types of structures have also been observed in some natural minerals. Under fast crystal growth conditions, individual crystals split to form a series of sub‐individual NWs with sheaf‐like or spherulite structures. The orthorhombic Bi_2_S_3_ structure is composed of chain‐like structures with stoichiometric compositions along the c‐axis, and the binding of these chains occurs in the [010] direction. Owing to weak binding between chains, crystal splitting is favorable for the formation of sheaf‐like NW structures.^[^ ^53^ ^]^ Similarly, BiSBr forms an orthorhombic crystal structure with a double‐chain structure of [(BiSX)_2_]_n_ bound by van der Waals forces.^[^ ^54^ ^]^ Therefore, crystal splitting of BiSBr NWs may also be favorable due to the weak van der Waals interaction between chains, leading to sheaf‐like NBs. Because DT surfactants were added to the reaction mixture, the large surface areas of NBs after crystal splitting were stabilized by the adhesion of DT ligands to the NB surfaces, resulting in thermodynamically stable sheaf‐like NBs.

From the viewpoint of nucleation and growth theory, the NW bundling mechanism can also be described as non‐crystallographic branching or splitting from the nucleus.^[^ ^55^, ^56^ ^]^ In this reaction, the crystalline seeds aggregate owing to the presence of binding agents, such as polyvinylpyrrolidone^[^ ^57^ ^]^ and glucose,^[^ ^58^ ^]^ or to minimize their surface area. Then, crystalline NWs are grown from these aggregated seeds via successive branching to form sheaf‐like or spherulite nanostructures. In the reaction investigated in our study, we also speculated that the reaction intermediates were produced in the early stages of the reaction and acted as crystalline nuclei for the growth of BiSBr NBs. However, no direct evidence of the growth of BiSBr NBs from crystalline nuclei was observed in the study. Nevertheless, we observed the formation of layered BiOBr aggregates, composed of multiple 2D nanoplatelets (NPs) with the mixture of BiSBr NWs and NBs, in an early stage of the reaction, at ≈90 °C, when ODE was used as the co‐solvent in the reaction (Figures S11 and S12, Supporting Information). As the reaction time increased, the BiOBr NPs completely disappeared over time. In a previous study, this seed‐mediated synthesis of chalcogenide NCs (particularly, Bi_2_S_3_ NCs) from oxychloride was accomplished using BiOCl as the oxychloride seeds,^[^ ^59^ ^]^ suggesting the possibility of utilizing oxyhalide nuclei for the growth of NCs. Additionally, we investigated a reaction in which BiOBr NPs were synthesized initially in the absence of S precursors and then transformed into BiSBr NBs via the addition of S precursors and the DT solvent. TEM and XRD analyses confirmed that the BiOBr NPs completely transformed into BiSBr NBs (Figure S13, Supporting Information). Although these experimental results may suggest that BiOBr NPs act as crystalline seeds for the formation of BiSBr NBs, additional microscopic analyses should be performed to capture the crystallographic interfaces between the seeds and bundles to corroborate the growth mechanism of BiSBr NBs.

### 2.6 Fabrication of Solution‐Processed Flexible Photodetectors

**Figure 5:**
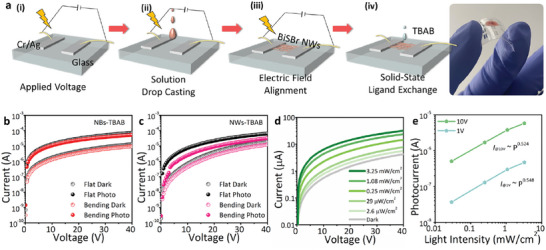
a) Schematic of the fabrication of photodetectors by sequential electric field alignment and solid‐state ligand exchange. Current (*I*)–voltage (*V*) characteristics of flexible photodetectors fabricated using b) BiSBr NWs and c) NBs before and after 0.8% strain application under dark and illumination conditions. Photocurrent characteristics were examined under 532 nm laser illumination at a light intensity of 0.7 mW cm^−^^2^. d) *I–V* curves of the BiSBr NB‐based flexible photoconductor under a strain of 0.8% under dark and 532 nm laser illumination conditions at different light intensities ranging from 0.0026 to 3.25 mW cm^−2^. e) Light intensity‐dependent photocurrent of the BiSBr NB‐based photoconductor under 0.8% strain and 532 nm illumination at 1 and 10 V.

Owing to their 1D nanostructures with high aspect ratios, flexibilities, and colloidal stabilities, BiSBr NWs and NBs were easily integrated into optoelectronic devices. High colloidal stabilities enable uniform depositions of functional NCs on the substrate at room temperature, which are ideal for solution‐processed fabrication of photodetectors on flexible polymeric substrates.^[^ ^60^, ^61^ ^]^ The photodetectors were constructed EFA process^[^ ^24^, ^62^ ^]^ followed by solid‐state ligand exchange,^[^ ^63^, ^64^, ^65^, ^66^ ^]^ as described in **Figure** **5**a. For EFA, a 10 µL solution of BiSBr NWs or NBs was drop‐casted on pre‐patterned electrodes on polyethylene terephthalate (PET) substrates, and then, NWs and NBs filled the space between two electrodes because their internal polarizations resulted in a strong interconnection between BiSBr NWs and NBs (Figure S14, Supporting Information). Solid‐state ligand exchange was conducted using tetrabutylammonium bromide (TBAB) to strip the original ligands, thus enhancing the electrical coupling between NWs and NBs. FTIR spectra obtained before and after ligand exchange demonstrated complete removal of original DT ligands from the BiSBr NW and NB surfaces (Figure S15, Supporting Information).

Figure 5b,c depicts the current characteristics of BiSBr NW and NB films under dark and illuminated conditions at a wavelength of 532 nm and a light intensity of 0.7 mW cm^−2^, respectively. Enhanced current under illumination suggested the abilities of BiSBr NW and NB films to detect photons with a wavelength of 532 nm, which is higher than the bandgaps of BiSBr NWs and NBs. Responsivities and EQEs of BiSBr NW and NB films were 18.64 and 25.15 A W^−1^ and 4,344 and 5,863%, respectively, on the flat surface without strain. These values were substantially higher than those of the films obtained before ligand exchange (Figure S16, Supporting Information); this finding was ascribed to efficient charge transport between NBs and NWs after stripping the insulating surface ligands. These bulkier BiSBr NBs exhibited inferior photoresponsivities and EQEs to those of BiSBr NWs and BiSBr NBs synthesized using 90 vol% ODE (Figure S17, Supporting Information). Responsivities and EQEs of the BiSBr NBs prepared using ODE:DT ratios of 70:30 and 10:90 were 10.45 and 0.31 A W^−1^ and 2,436 and 72%, respectively. This result might be ascribed to the inadequate colloidal stability of bulky BiSBr, which deteriorated the uniform depositions of active layers. BiSBr NBs synthesized using an ODE:DT volume ratio of 40:60 at 120 °C and all NBs constructed at 150 °C were not colloidally stable, and the corresponding films demonstrated almost no electrical properties due to the difficulty in the formation of uniform films.

Under 0.8% strain, the responsivity and EQE of the BiSBr NW‐based photoconductor decreased to 8.26 A W^−1^ and 1,924%, leading to a 56% reduction in performance after strain. In contrast, under 0.8% strain, the BiSBr NB‐based photoconductor exhibited a responsivity of 18.15 A W^−1^ and an EQE of 4,230%, resulting in a 28% decrease in performance after strain. These results indicated that the photoconductor fabricated using BiSBr NBs was more resilient to external strain. The reduction in responsivity under applied strain is attributed to the formation of cracks in the metal electrodes or an increase in the spacing between NWs or NBs, which degrades the collection efficiencies of photocarriers.^[^ ^67^, ^68^ ^]^ Figure 5d depicts *I–V* characteristics of the BiSBr NB‐based photoconductor under 0.8% strain in the light intensity range from 0.0026 to 3.25 mW cm^−^^2^. Under a strain of 0.8%, the flexible photoconductor demonstrated a degree of linearity across multiple orders of magnitude of illumination intensity. Via curve fitting of the data shown in Figure 5e using equation 3 (Experimental Section), the values of the exponent that determines the response of photocurrent to the light intensity (*n*) were evaluated to be 0.548 and 0.524 at 1 and 10 V, respectively. Non‐unity exponent of a photoconductor arises from an intricate sequence of events involving electron–hole generation, trapping, and recombination within the photoconductor.^[^ ^9^, ^48^, ^69^ ^]^ Table S2 (Supporting Information) provides an overview of the Bi‐material‐based photodetector performance. BiSBr NWs and NRs exhibit a high responsivity, which is comparable with or even exceeds the reported values. Moreover, our BiSBr NWs and NRs outperform the previously reported Bi‐based flexible photodetectors, despite being under strain. High photoresponsivity of the BiSBr NB‐based photoconductor on flexible substrates and solution processability with toxic element‐free colloidal semiconductors reveal unique potentials of BiSBr NBs for application in the development of novel optoelectronic devices.

## 3 Conclusion

In sum, this study demonstrated the injection‐free heat‐up synthesis of highly uniform colloidal 1D BiSX NWs and NBs. To this end, first, BiSBr NWs with high aspect ratios were synthesized using BiBr_3_ and S precursors in DT. Bundling of BiSBr NWs was achieved using a solvent mixture of ODE and DT. The degree of bundling was further tailored by changing the relative ODE:DT volume ratio. The number of BiSBr NWs in a single NB increased with a decrease in the volume fraction of ODE in the solvent mixture, altering the morphologies from sheaf‐like structures to spherulite nanostructures. 1D high‐aspect‐ratio BiSCl and BiSI NWs were also fabricated using BiCl_3_ and BiI_3_ precursors. For the synthesis of BiSI NWs, OA was used to fully dissolve the BiI_3_ precursors at low temperatures, thereby enabling the formation of 1D BiSI NWs under relatively low‐temperature conditions. BiSX NWs and NBs exhibited visible and near‐infrared absorptions from 1.91 eV (BiSCl), 1.88 eV (BiSBr), to 1.53 eV (BiSI). BiSBr NWs and NBs produced herein demonstrated excellent colloidal stabilities, enabling room‐temperature solution‐processed device fabrication on flexible substrates. DFT calculation revealed that the expansion of c‐lattice parameters and the blue shift of bandgap occurred due to the confined dimensionality of ultrathin BiSBr NWs. Visible‐light photodetectors were synthesized using BiSBr NWs and NBs via sequential EFA and solid‐state ligand exchanges in TBAB solution. Responsivities and EQEs of BiSBr NW and NB films were 18.64 and 25.15 A W^−1^ and 4,344 and 5,863%, respectively, under 532 nm illumination with a light intensity of 0.7 mW cm^−2^. This high performance was not significantly affected under strain on the flexible PET substrate. The proposed reaction requires relatively low temperatures (<150 °C) and short reaction times (<5 min) and involves a simple injection‐free process, which exhibits considerable potential for large‐scale mass production. Thus, the novel method for synthesizing 1D colloidal BiSX NWs and NBs comprising nontoxic elements serves as a guideline for designing highly efficient, flexible, and environmentally friendly electronic and optoelectronic devices.

## 4 Experimental Section

### Materials

Bismuth(III) bromide (BiBr_3_, 98%), bismuth(III) chloride (BiCl_3_, 98%), bismuth(III) iodide (BiI_3_, 99%), sulfur (S, 99.98%), 1‐octadecene (ODE, technical grade, 90%), oleic acid (OA, technical grade, 90%), and 1‐dodecanethiol (DT, 98%) were purchased from Sigma‐Aldrich. All chemicals were used as received without further purification.

### Synthesis of BiSBr NWs

In a three‐necked flask, 0.1012 g of BiBr_3_ and 0.025 g of S were added to 50 mL of DT. The reaction mixture was stirred under vacuum at 90 °C for 90 min. Then, the resulting solution was heated to 120 °C followed by reaction for 3 min. The resulting material was purified by adding toluene followed by centrifugation at 6000 rpm for 3 min. The acquired precipitates were dispersed in toluene to obtain colloidal BiSBr NWs.

### Large‐Scale Synthesis of BiSBr NWs

In a three‐necked flask, 600 mL of DT was added and stirred under a vacuum at 125 °C for 3 h. Then, 125 g of BiBr_3_ and 31 g of S were added to the reaction mixture and stirred under vacuum at 80 °C for 3 h. The reaction mixture was heated to 120 °C and maintained for 3 min for the formation of BiSBr NWs. The resulting material was purified by adding toluene, followed by centrifugation at 6000 rpm for 3 min.

### Synthesis of BiSBr NBs

For preparing BiSBr NBs, the procedure for the synthesis of BiSBr NWs was followed except for the use of a solvent mixture of ODE and DT.

### Synthesis of BiSCl NWs

In a three‐necked flask, 0.0711 g BiCl_3_ and 0.025 g S were introduced into a mixture of 35 ODE and 15 mL DT. The reaction mixture was stirred under vacuum at 90 °C for 90 min. Thereafter, the resulting solution was heated to 140 °C followed by a reaction for 10 min. The resulting material was purified using the same procedure as that employed for BiSBr NWs.

### Preparation of BiSI NWs

In a three‐necked flask, 0.075 g BiCl_3_ and 0.05 g S were added to a mixture of 10 mL ODE, 10 mL DT, and 40 mL OA. The reaction mixture was stirred under vacuum at 90 °C for 90 min. Then, the solution was heated to 170 °C followed by a reaction for 15 min. The resulting material was purified using the same procedure as that employed for BiSBr NWs.

### Device Preparation

Cr and Ag were deposited on glass and PET insulating substrates with thicknesses of 5 and 80 nm, respectively, using a thermal evaporator. For EFA, 10 µL BiSBr NW or NB dispersion in toluene was drop‐casted between electrodes, and a voltage of 20 V was applied for 30 s. Subsequently, solid‐state ligand exchange was performed by treating BiSBr NW and NB films with 30 mm TBAB ligand solution for 1 min. The resulting films were rinsed three times with methanol.

### Characterization

SEM images were obtained using Carl Zeiss SIGMA. TEM, HRTEM, scanning TEM (STEM), and EDS were conducted using a JEOL JEM‐F200 system operating at 200 kV. XRD was performed using a Bruker‐AXS New D8‐Advance system. Diffuse reflectance spectra were acquired using a JASCO V‐770 spectrometer. The Kubelka–Munk function was expressed as *F*(*R*) = (1‐*R*)^2^/2*R*, where *R* was the reflectance of the infinitely thick samples; the band gap can be determined by the following equation: (*F*(*R*)*hν*)^n^ = *A*(*hν*−*E*_g_), where *n* = 1/2 and 2 for indirect and direct transitions, respectively.^[^ ^70^ ^]^ XPS was conducted using a Thermo Fisher Scientific K‐Alpha system. *I–V* characteristics of photodetectors were measured using a source meter (Keithley 2400, Tektronix) with a 532 nm laser (MLL‐III‐532/150 mW, Changchun New Inc.). Responsivity (*R*) and EQE were calculated using the following equations:

(1) $$R=\frac{I_{ph}}{P_{\lambda }A}$$

(2) $$EQE\;=\;R\frac{h\cdot c}{\lambda \cdot q}$$

where *I*_ph_ was the photocurrent, *P*_λ_ was the power of at the illumination wavelength (mW cm^−2^), A was the sample area (channel area between two electrodes), *h* = 6.63 × 10^–34^ J s was the Plank's constant, *c* = 3 × 10^8^ m s^−1^ was the light velocity, and *q* = 1.6 × 10^–19^ C was the elementary charge. The nonlinear relationship between the change in photocurrent and light intensity follows a power law.^[^ ^71^ ^]^

(3) $$I_{\mathrm{ph}}=AP^{n}$$

where *A* was a constant for an illumination wavelength and the exponent *n* (0.5 < *n* < 1) determines the response of the photocurrent to light intensity.

If shot noise predominates as the primary source of noise in a photodetector, then the specific detectivity, denoted as D*, can be expressed as:

(4) $$D^{*}=R\;x\;\frac{\sqrt{A}}{\sqrt{2qI_{d}}}$$

where *I*_d_ was the dark current of the device.

### Computational Details

The Vienna ab initio simulation package (VASP) was used to perform first‐principles DFT calculation based on the projector augmented‐wave pseudopotential,^[^ ^72^, ^73^ ^]^ within the Perdew–Burke‐Ernzerhof (PBE) generalized gradient approximation (GGA).^[^ ^74^ ^]^ The internal atomic positions and lattice parameters were fully optimized with a force criterion of 0.01 eV Å^−1^. In the case of strained 1 × 1 chain BiSBr, only the internal atomic positions were optimized at the fixed chain lattice values, where the strain ε along the chain direction (z‐direction) varies from −1.5% to 1%. To account for the interchain van der Waals interactions, the non‐local optB86b‐vdW functional was used for all the structure optimizations.^[^ ^75^ ^]^ The calculation was also tested using PBEsol GGA functional^[^ ^76^ ^]^ and found no qualitative differences in terms of the band gap, c‐lattice parameter, or any other major electronic detail. For both 1 × 1 and 2 × 2 chain BiSBr, a vacuum thickness of >15 Å was taken along the direction perpendicular to the chain to avoid any artificial interaction between the periodic images. An energy cutoff of 500 eV as well as 1 × 1 × 12 and 6 × 6 × 12 *k* points were used for the different chains and bulk BiSBr, respectively. It should be noted that although GGA suffers from the well‐known band‐gap underestimation, the neglection of spin–orbit coupling (SOC) leads to an overestimation of the band gap, mainly due to the significant proportion of heavy Bi element in the conduction band.^[^ ^32^ ^]^ Thus, these two effects compensate each other in BiSBr, resulting in a similar band gap as that obtained from hybrid Heyd–Scuseria–Ernzerhof functional including SOC (HSE+SOC) as shown in a previous study.^[^ ^32^ ^]^ For bulk BiSBr, different functionals were used in previous computational studies, reporting both direct and indirect band gaps: the Γ to Γ direct gap from HSE+SOC,^[^ ^32^ ^]^ the Γ‐Z to Y‐S indirect gap from PBE with Tkatchenko–Scheffler correction^[^ ^36^ ^]^ and PBEsol,^[^ ^77^ ^]^ and the Γ‐Z to Γ‐Y indirect gap from r^2^SCAN.^[^ ^78^ ^]^ The result of bulk BiSBr was therefore consistent with refs. [36, 74, 75], but not with ref. [32]. It should be noted that the Γ‐S line was also considered and found that the Γ‐Z to Γ‐S indirect gap was smaller by ≈0.07 eV than the previously reported Γ‐Z to Γ‐Y or Γ‐Z to Y‐S indirect gaps. This line was omitted in previous theoretical studies. The resulting difference (≈0.07 eV) was much smaller than the gap size itself (≈1.90 eV). To estimate the exciton Bohr radius ($a_{B}^{*}$) of bulk BiSBr, previously reported static dielectric constant and effective masses were used.^[^ ^79^, ^80^ ^]^ The averaged value of the dielectric constant ε_*avg*_ = Tr[ε]/3  ≈ 6.55 was computed from the *GW* results reported in Ref. [79], and the reduced mass 𝜇 ≈ 0.23 *m_e_* (where *m_e_* was the electron mass) was extracted from the values reported in Ref. [80]. The estimated exciton Bohr radius ($a_{B}^{*}$) determined using these parameters was $a_{B}^{*}=\varepsilon_{avg}m_{e}a_{B}/\mu \approx 1.5\;nm$, where *a_B_* was the Bohr radius. If other values of 𝜀 and 𝜇 extracted from the PBE results were adopted, the estimated $a_{B}^{*}$ was 4–8 nm, which was also comparable with the thickness value of the BiSBr NWs.^[^ ^31^, ^70^, ^73^ ^]^

## Conflict of Interest

The authors declare no conflict of interest.

## Supporting information

Supporting Information

## Data Availability

The data that support the findings of this study are available from the corresponding author upon reasonable request.
