# Supplementary material for: Ultrathin, High‐Aspect‐Ratio Bismuth Sulfohalide Nanowire Bundles for Solution‐Processed Flexible Photodetectors
Source: Adv Sci (Weinh). 2024 Jul 4;11(33):2403463. doi: 10.1002/advs.202403463 (PMC11434017; doi:10.1002/advs.202403463)
Supplement: Supplementary file 1 — Supporting Information [file ADVS-11-2403463-s001.docx]

Supporting Information

Ultrathin, High-Aspect-Ratio Bismuth Sulfohalide Nanowire Bundles for Solution-Processed Flexible Photodetectors

Da Won Lee, Seongkeun Oh, Dong Hyun David Lee, Ho Young Woo, Junhyuk Ahn, Seung Hyeon Kim, Byung Ku Jung, Yoonjoo Choi , Dagam Kim, Mi Yeon Yu, Chun Gwon Park, Hongseok Yun, Tae-Hyung Kim, Myung Joon Han*, Soong Ju Oh*, and Taejong Paik*


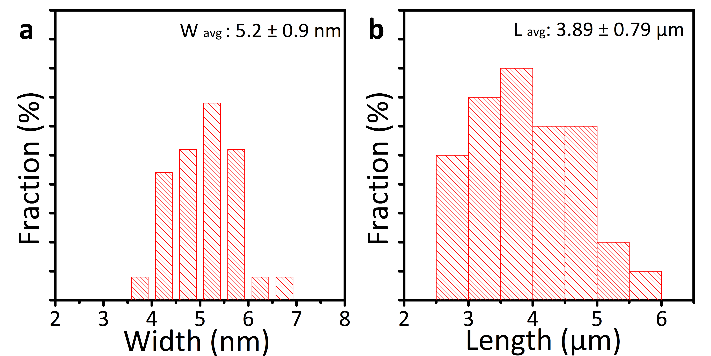


**Figure S1**. Size distribution histograms of BiSBr NWs with an average (a) width of 5.2 ± 0.9 nm and (b) length of 3.89 ± 0.79 μm.


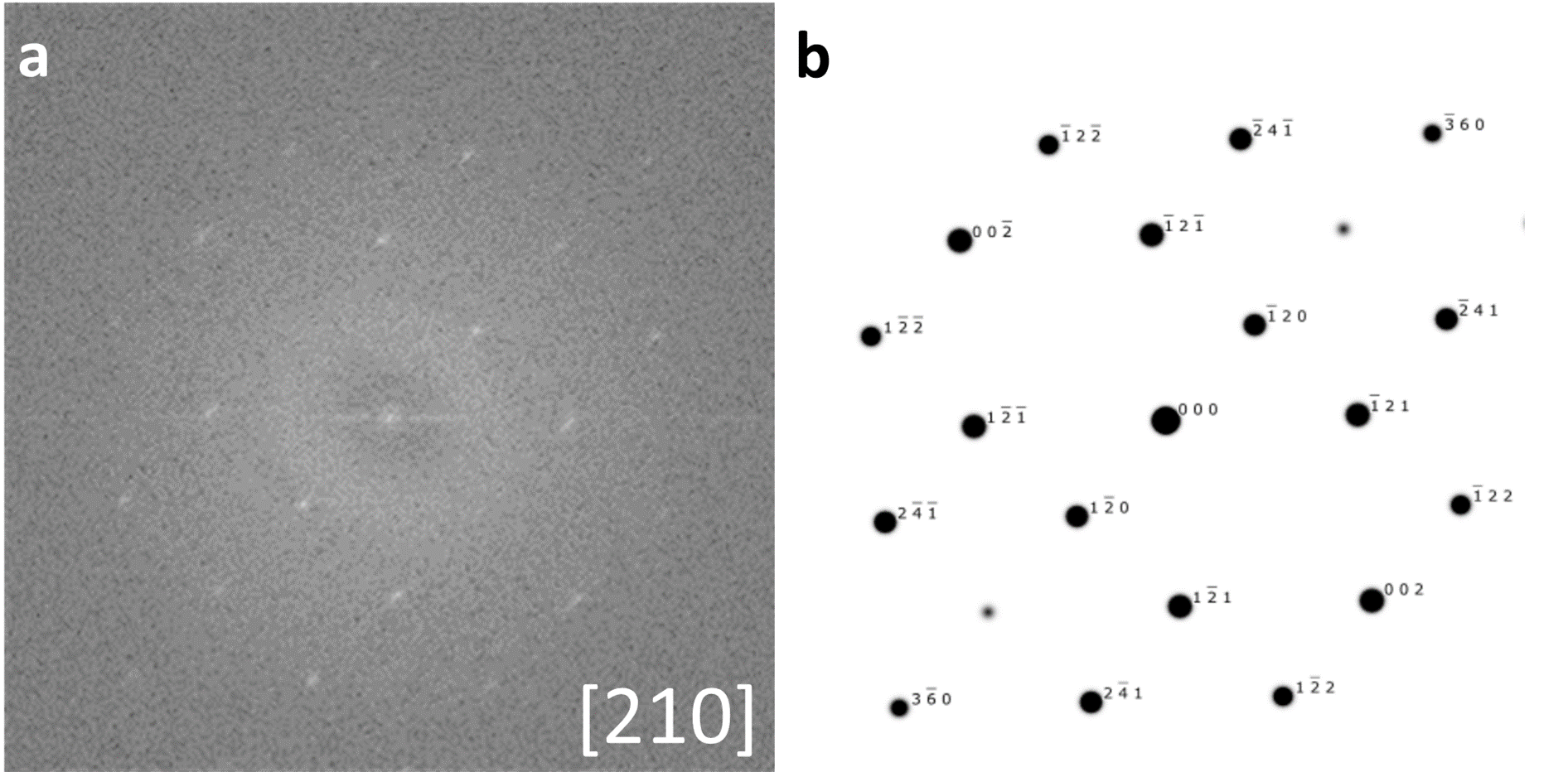


**Figure S2**. a) FFT of the HRTEM image and b) simulated ED pattern for a single BiSBr NW.


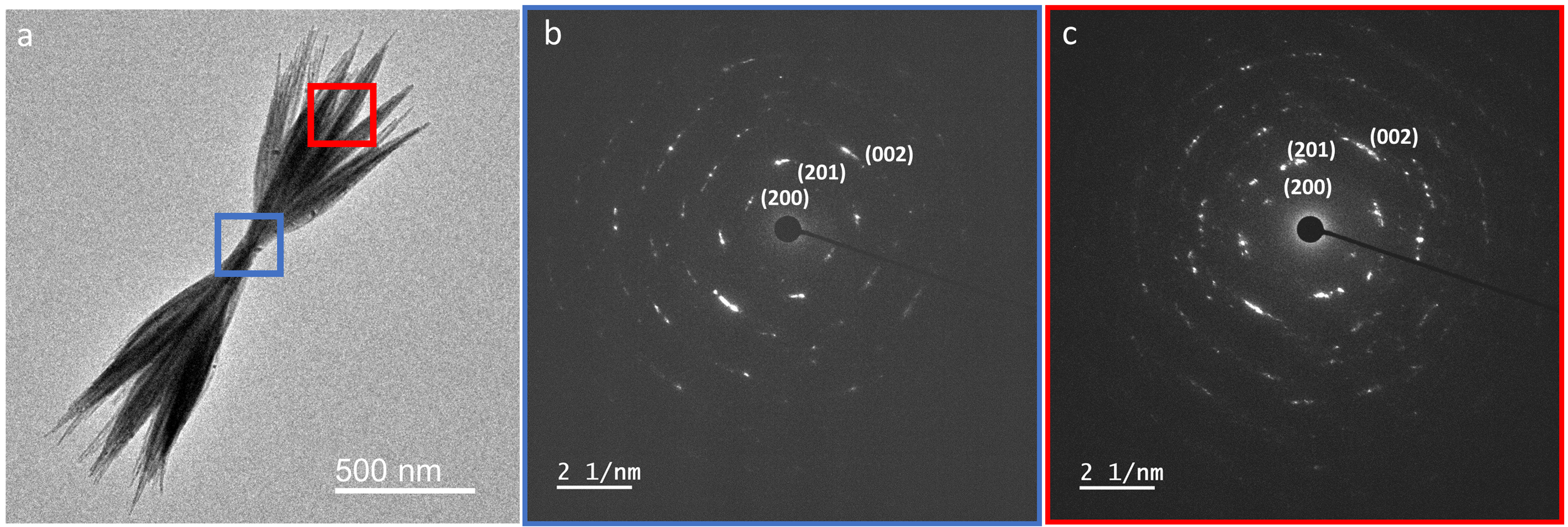


**Figure S3** a) TEM image of a BiSBr NB and selected area electron diffraction (SAED) patterns of BiSBr NB at the b) center and c) edge.


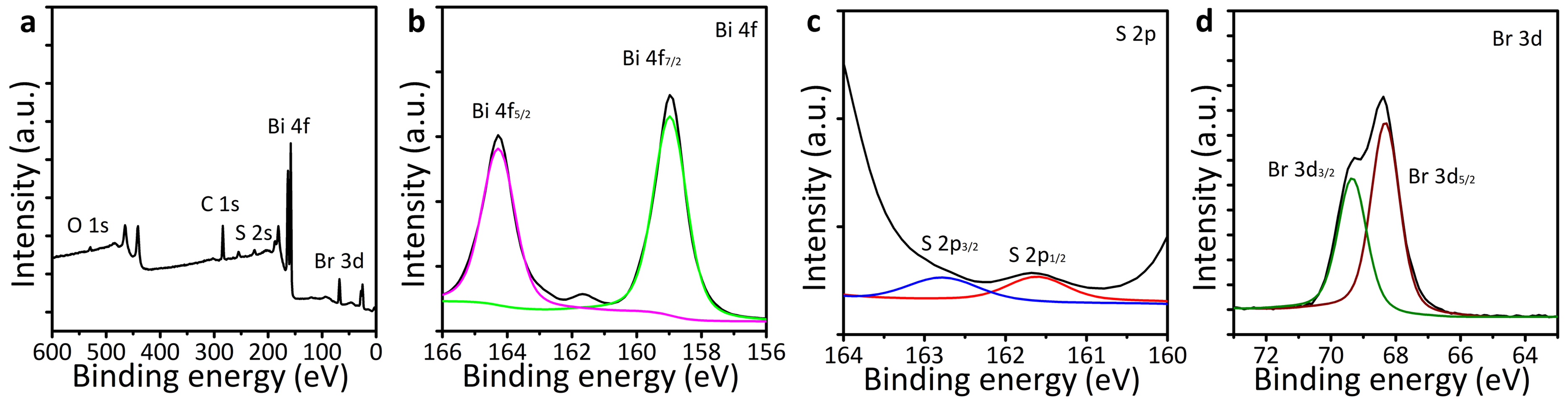


**Figure S4**. a) XPS full survey and high-resolution XPS profiles of b) Bi 4f, c) S 2p, and d) Br 3d.


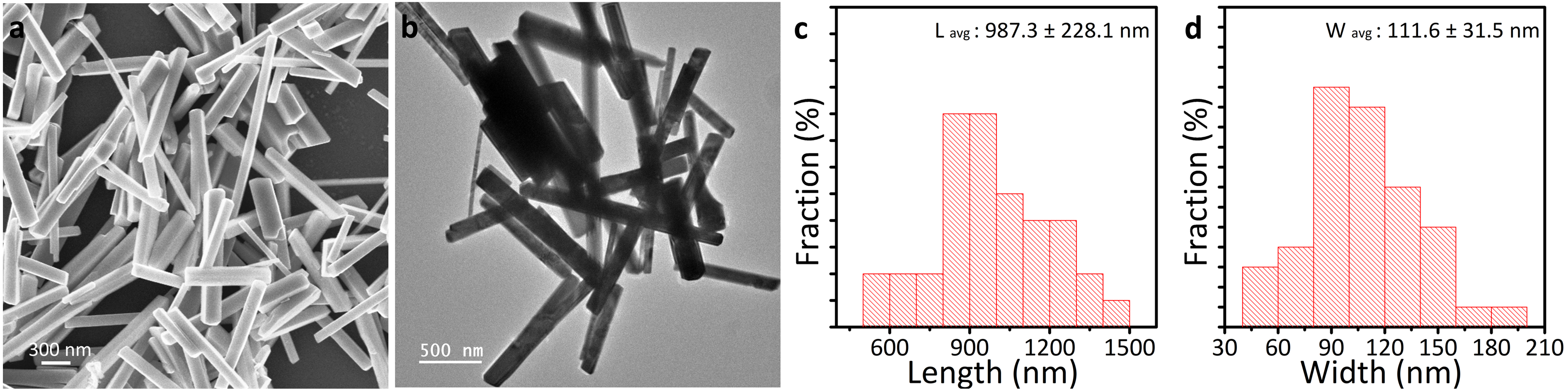


**Figure S5.** a) SEM and b) TEM images of BiSBr NRs synthesized using 100 vol% ODE and corresponding size histograms of c) length and d) width.


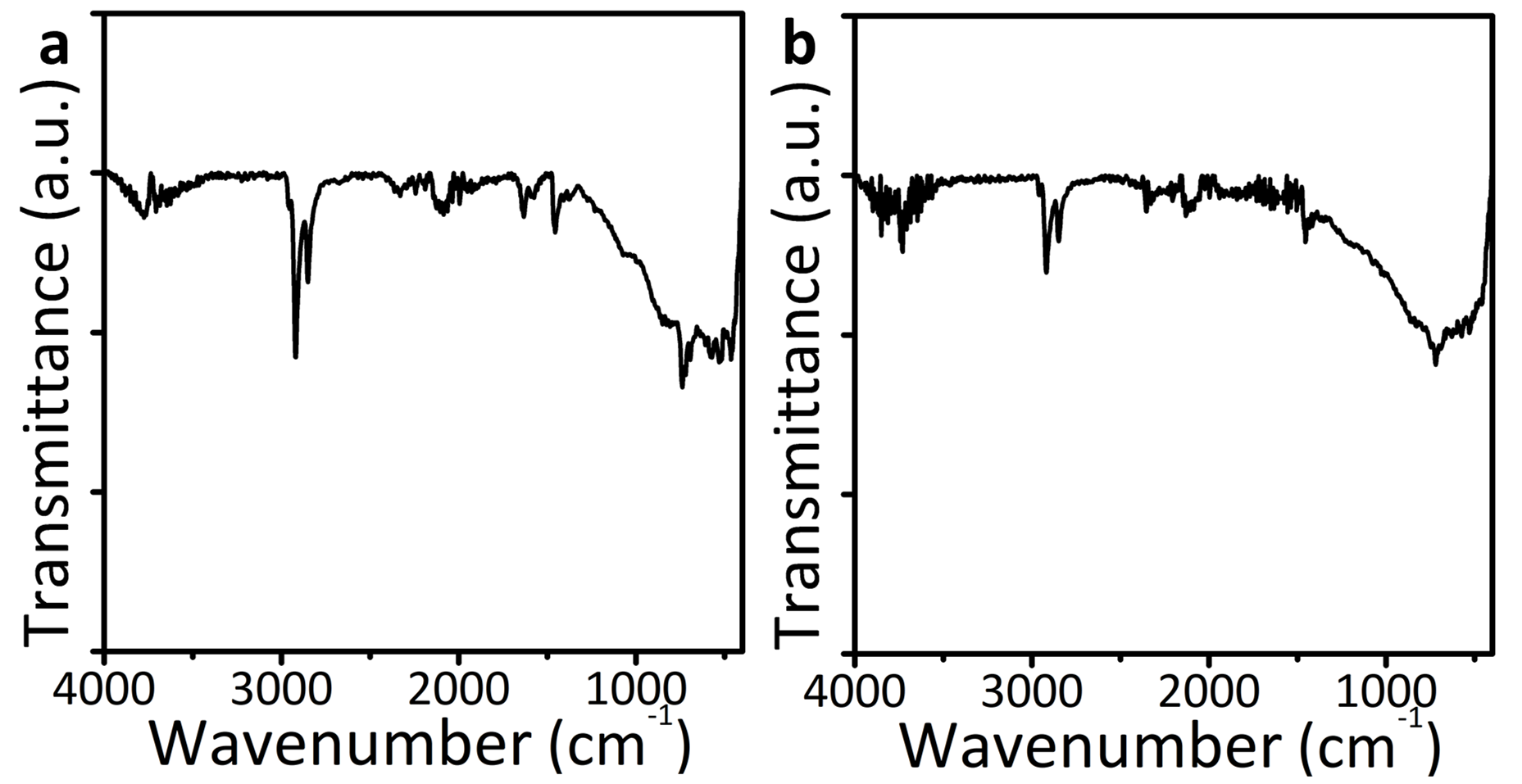


**Figure S6.** FT-IR spectra of a) BiSBr NWs and b) BiSBr NBs.

**
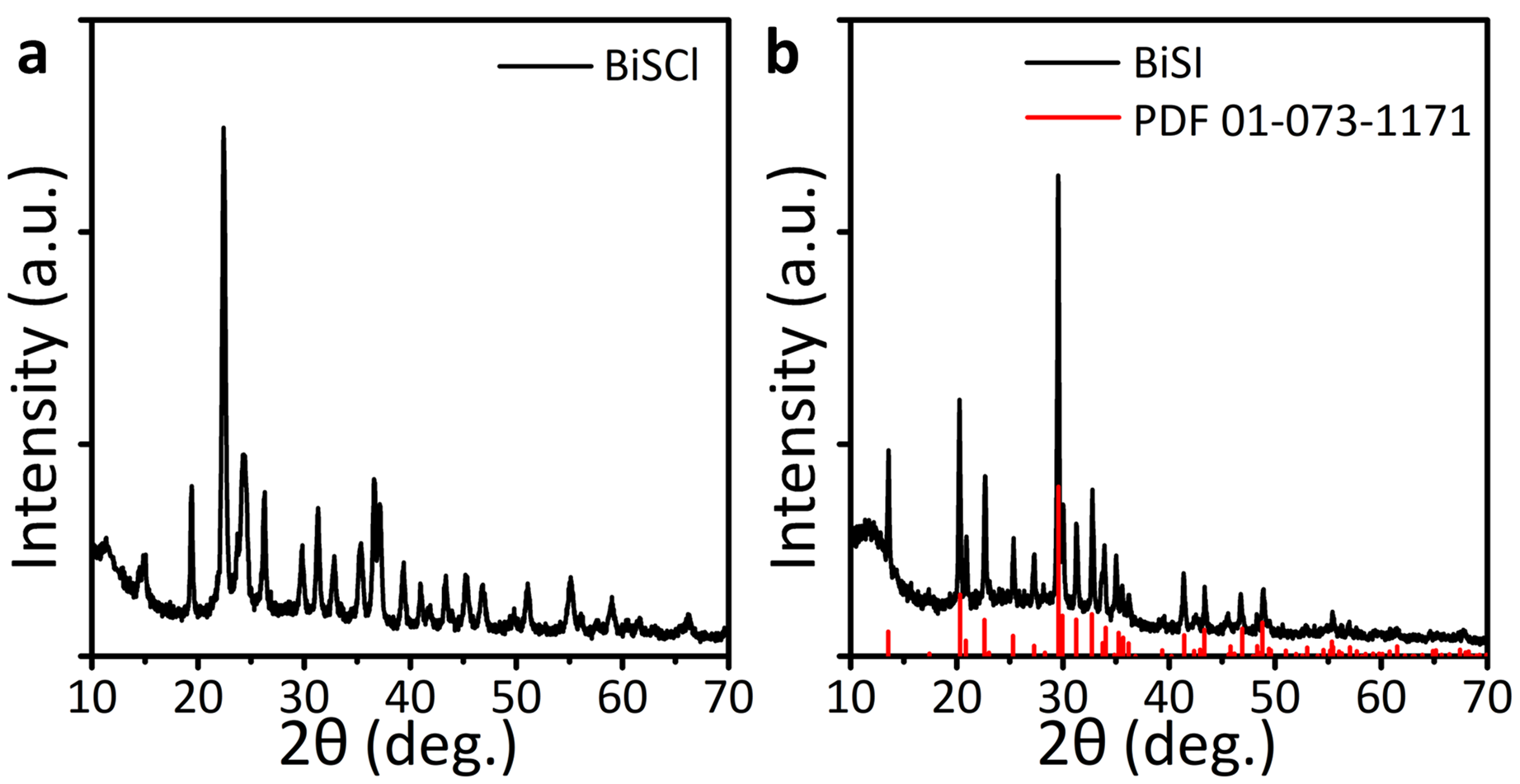
**

**Figure S7.** XRD patterns of BiSCl and BiSI NWs.


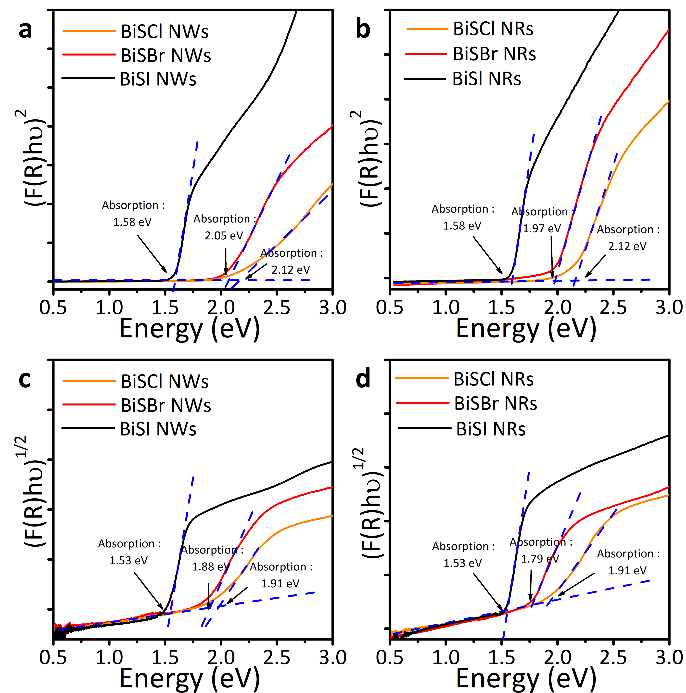


**Figure S8.** Kubelka–Munk plots for the direct transition of a) BiSX NWs and b) BiSX NRs, and for the indirect transition of c) BiSX NWs and d) BiSX NRs; here, X represents Cl, Br, and I.


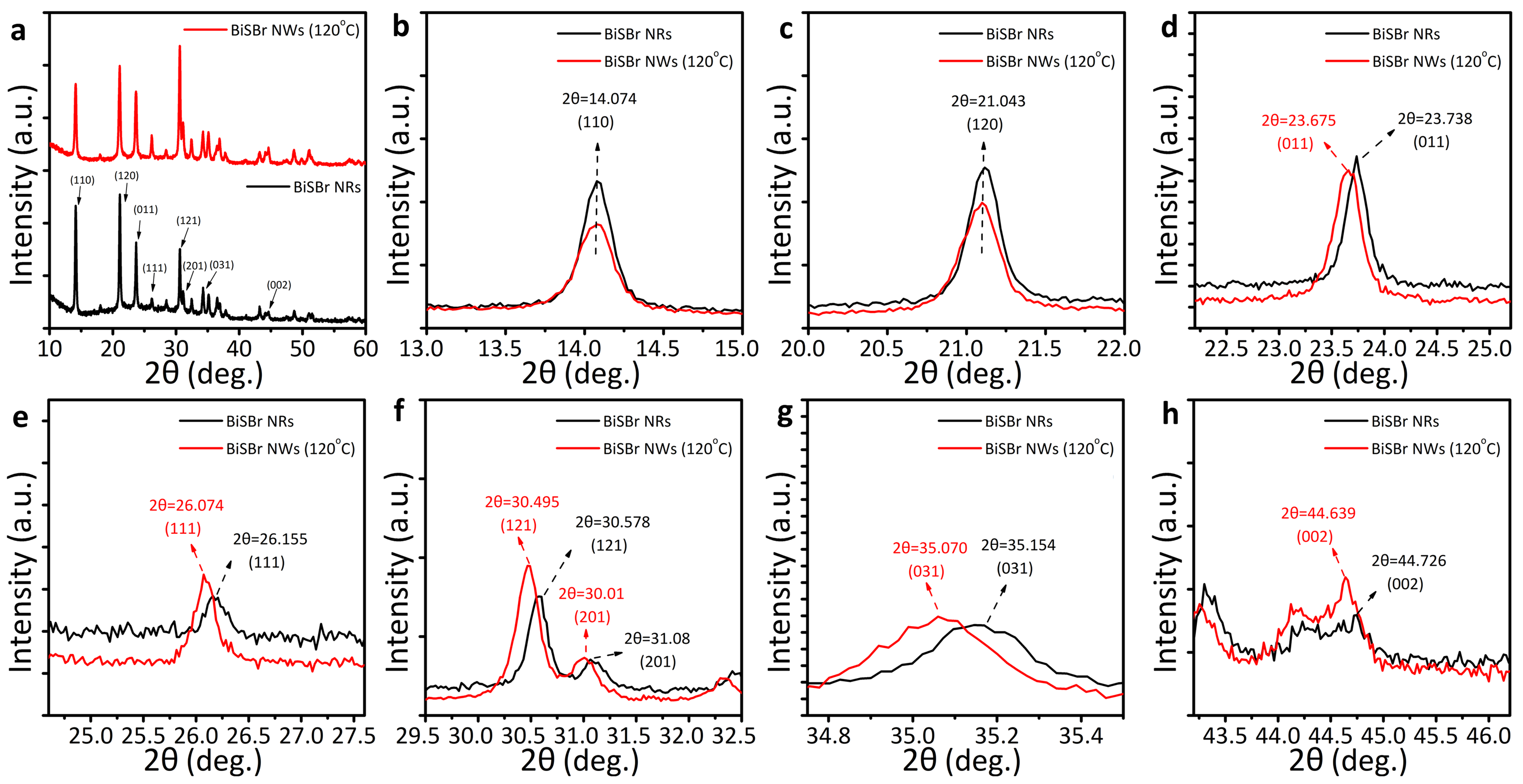


**Figure S9.** a) Full profiles of the XRD patterns of BiSBr NWs and NRs and selected peaks of b) (110), c) (120), d) (011), e) (111), f) (121) and (201), g) (031), and h) (002) diffractions.


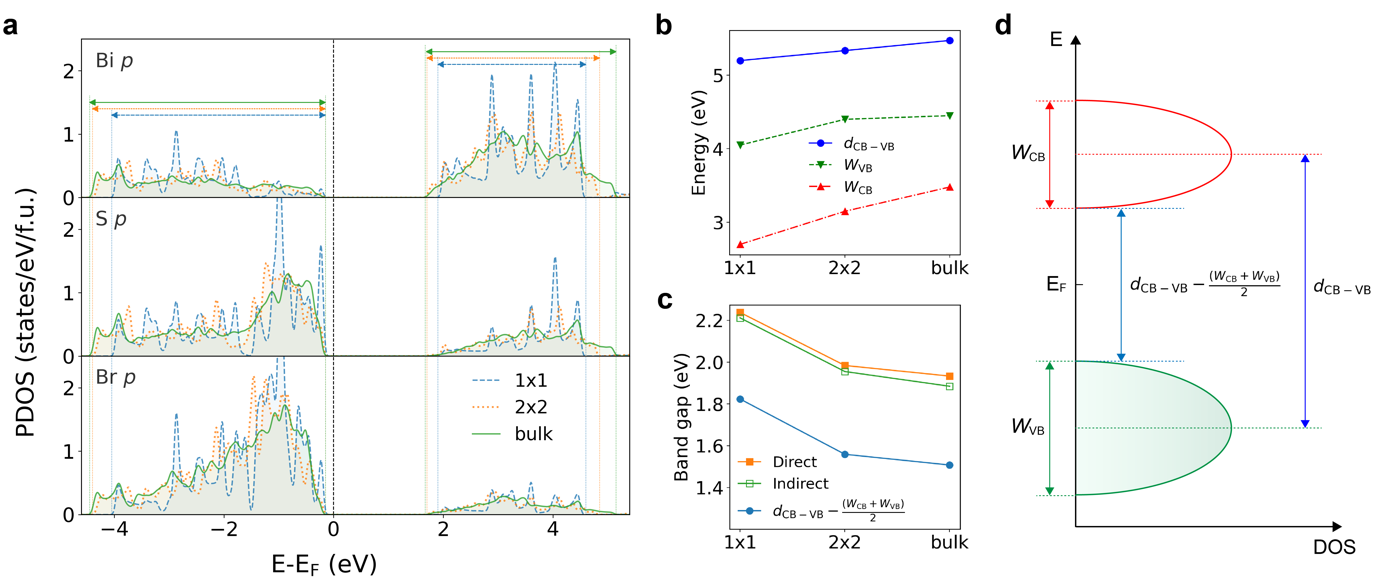


**Figure S10.** **a)** Calculated PDOS of Bi-p (top panel), S-p (middle panel), and Br-p (bottom panel) for 1 × 1 chain, 2 × 2 chain, and bulk BiSBr. The horizontal bidirectional arrows indicate the bandwidths of the VB and CB for 1 × 1 (dashed blue), 2 × 2 (dotted orange), and bulk (green solid). **b)** Calculated energy distance between the center of mass positions of the CB and VB ($d_{CB-VB}$; blue circles); the calculated bandwidths of the VB ($W_{\mathrm{VB}}$; green inverted triangles) and CB ($W_{\mathrm{CB}}$; red triangles) have been shown. **c)** Direct (orange squares) and indirect (green empty squares) gaps, derived from the DFT-calculated band structures, are compared with the modeling band gaps, which are estimated from the band-center distance and bandwidth (blue circles). **d)** Schematic representing the relationship between the model band gap and bandwidths shown in **b)** and **c)**. The Fermi level is denoted by $E_{F}$.

| Materials | Thickness | Direct E_g_ (eV) | Indirect E_g_ (eV) |
| --- | --- | --- | --- |
| BiSCl | 1 x 1  2 x 2  Bulk | 2.304  2.009  1.875 | 2.270  1.974  1.783 |
| BiSBr | 1 x 1  2 x 2  Bulk | 2.238  1.984  1.933 | 2.212  1.953  1.885 |
| BiSI | 1 x 1  2 x 2  Bulk | 2.179  1.808  1.743 | 2.149  1.775  1.707 |

**Table S1.** Calculated results of the thickness-dependent band gaps for BiSX (X = Cl, Br, I). The same increasing trend of both direct and indirect gaps is observed for all X as the thickness is reduced from the bulk to the 1$\times$1 chain.


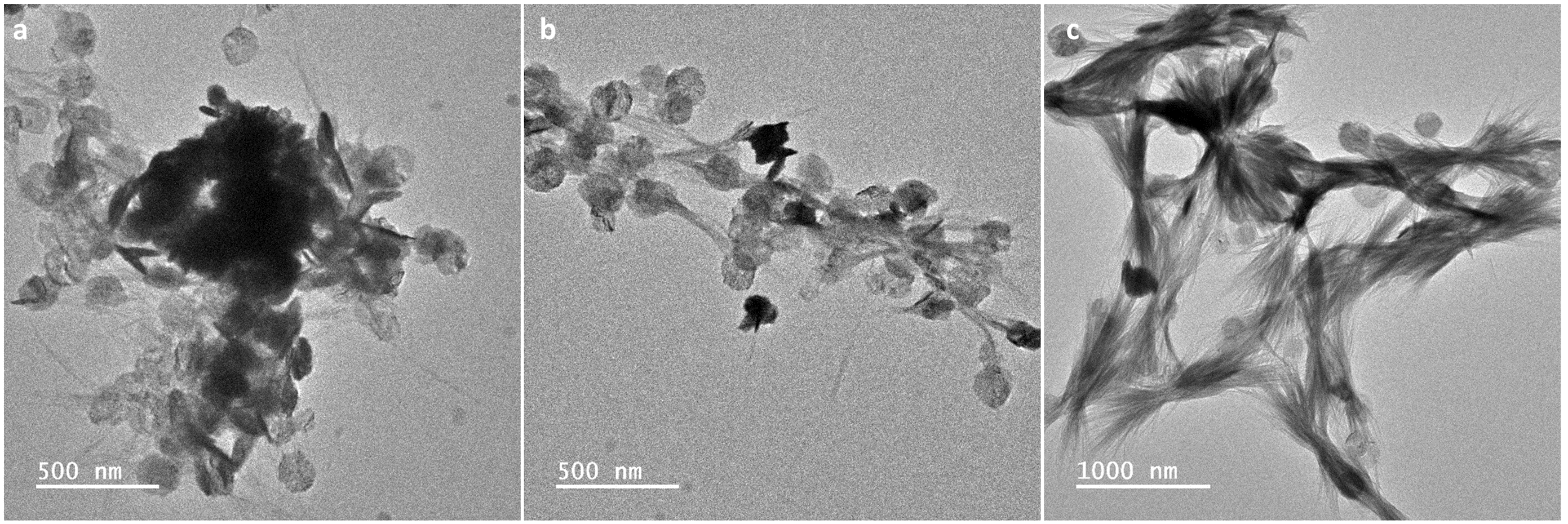
**Figure S11**. TEM images of the reaction intermediates formed at 90 °C


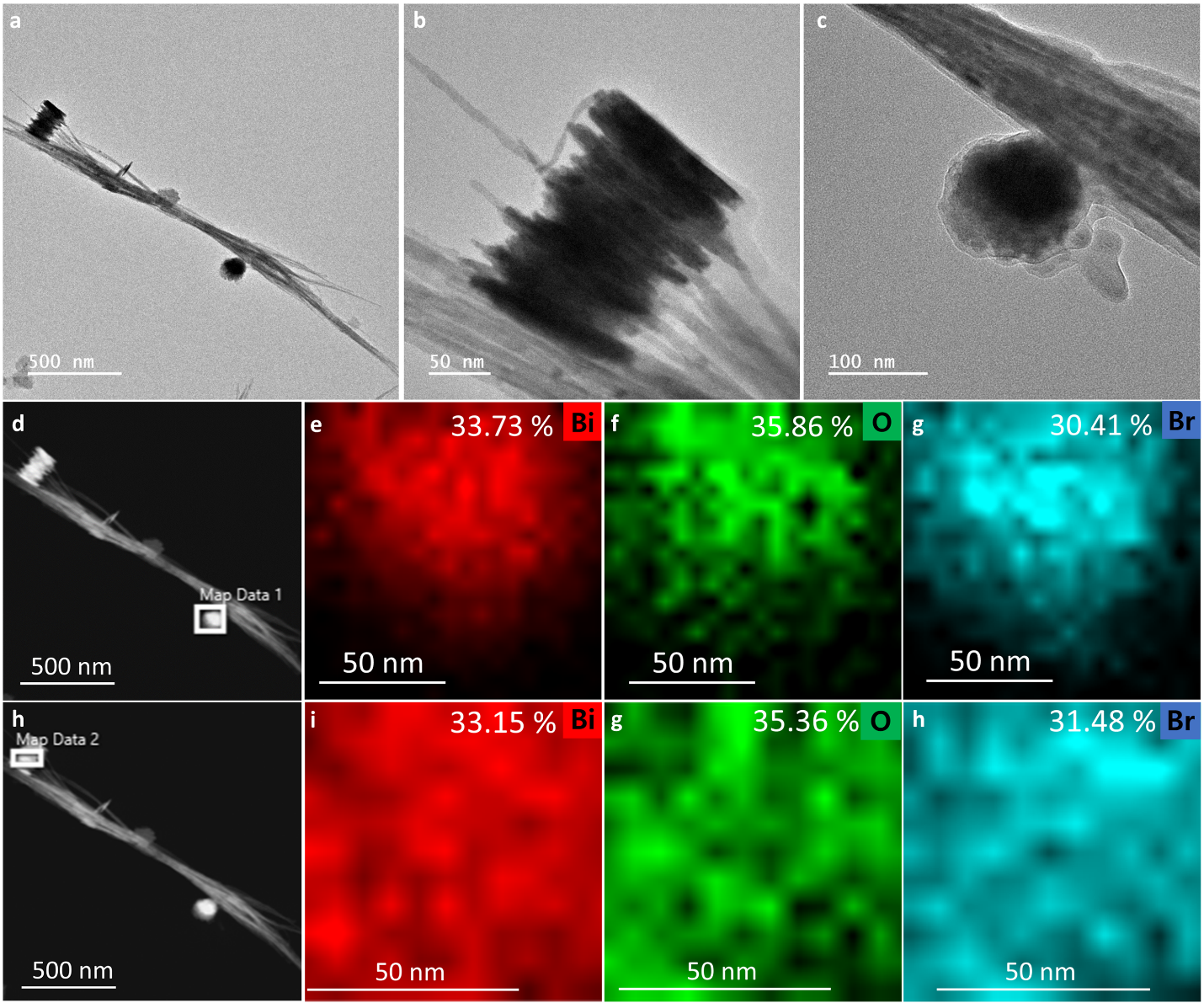
 **Figure S12**. a–c) TEM images of the reaction intermediates formed at 90 °C. d and h) STEM and elemental mapping images of selected regions: e and i) Bi, f and g) O, and g and h) Br atoms.


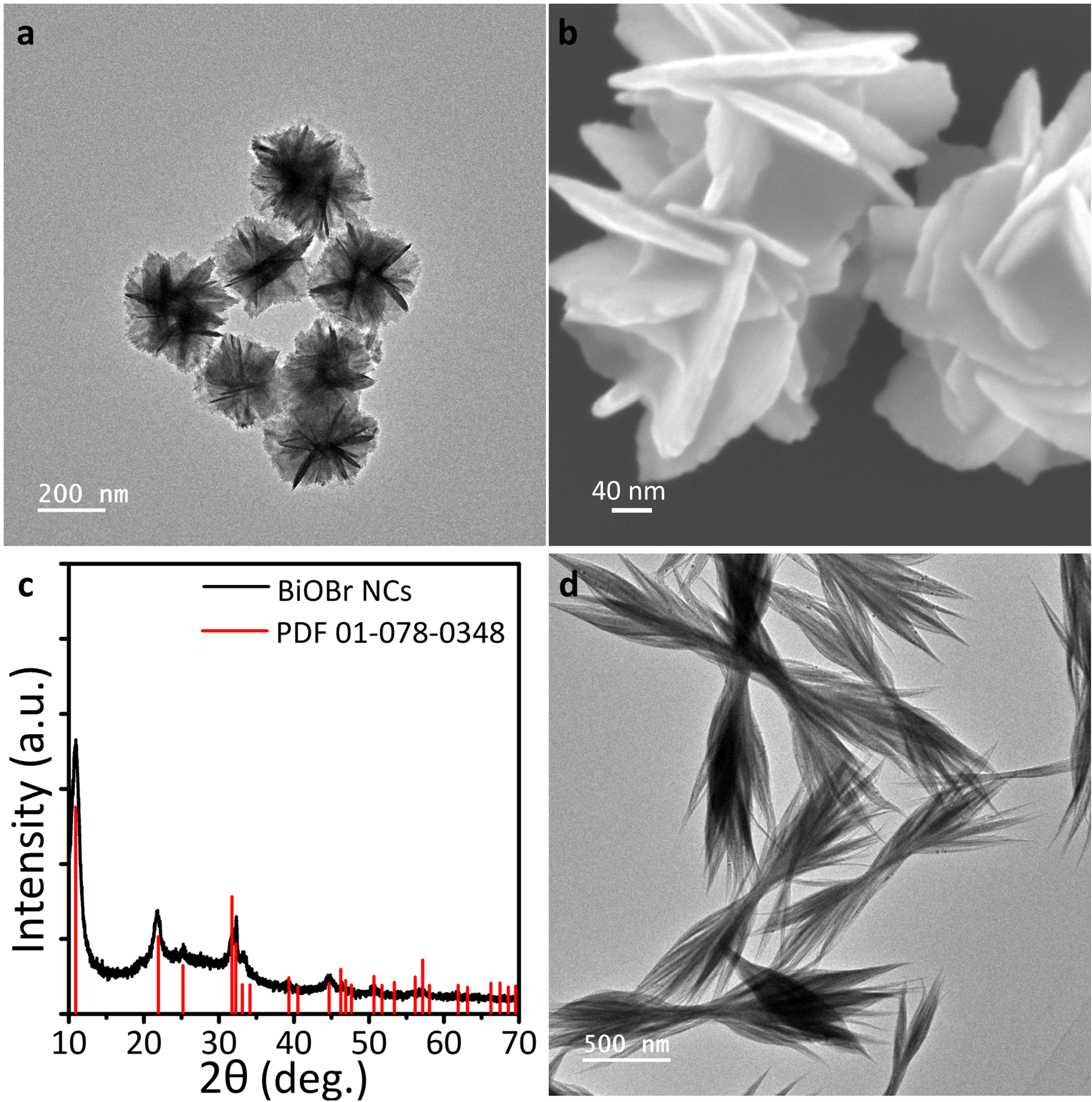


**Figure S13**. a–c) TEM images of the reaction intermediates formed at 90 °C. d and h) STEM and elemental mapping images of selected regions: e and i) Bi, f and g) O, and g and h) Br atoms.


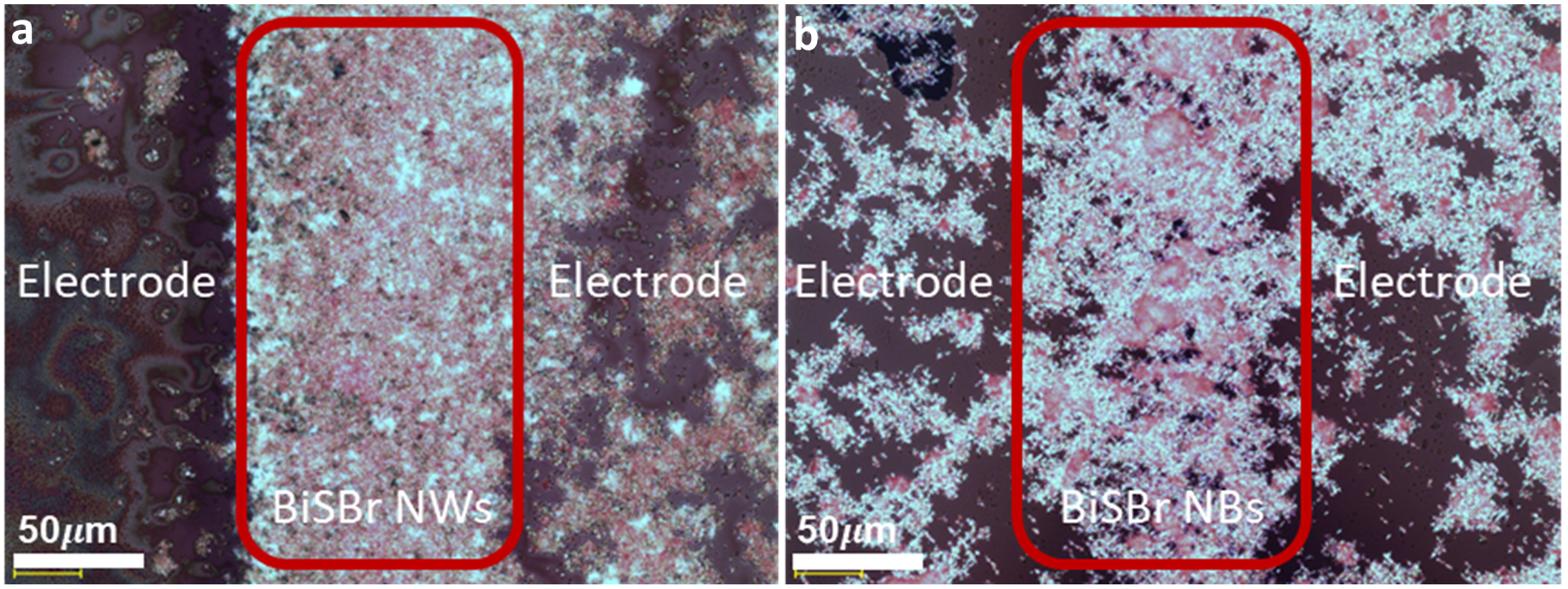


**Figure S14.** Images of the devices after electric field deposition of a) BiSBr NWs and b) BiSBr NBs.


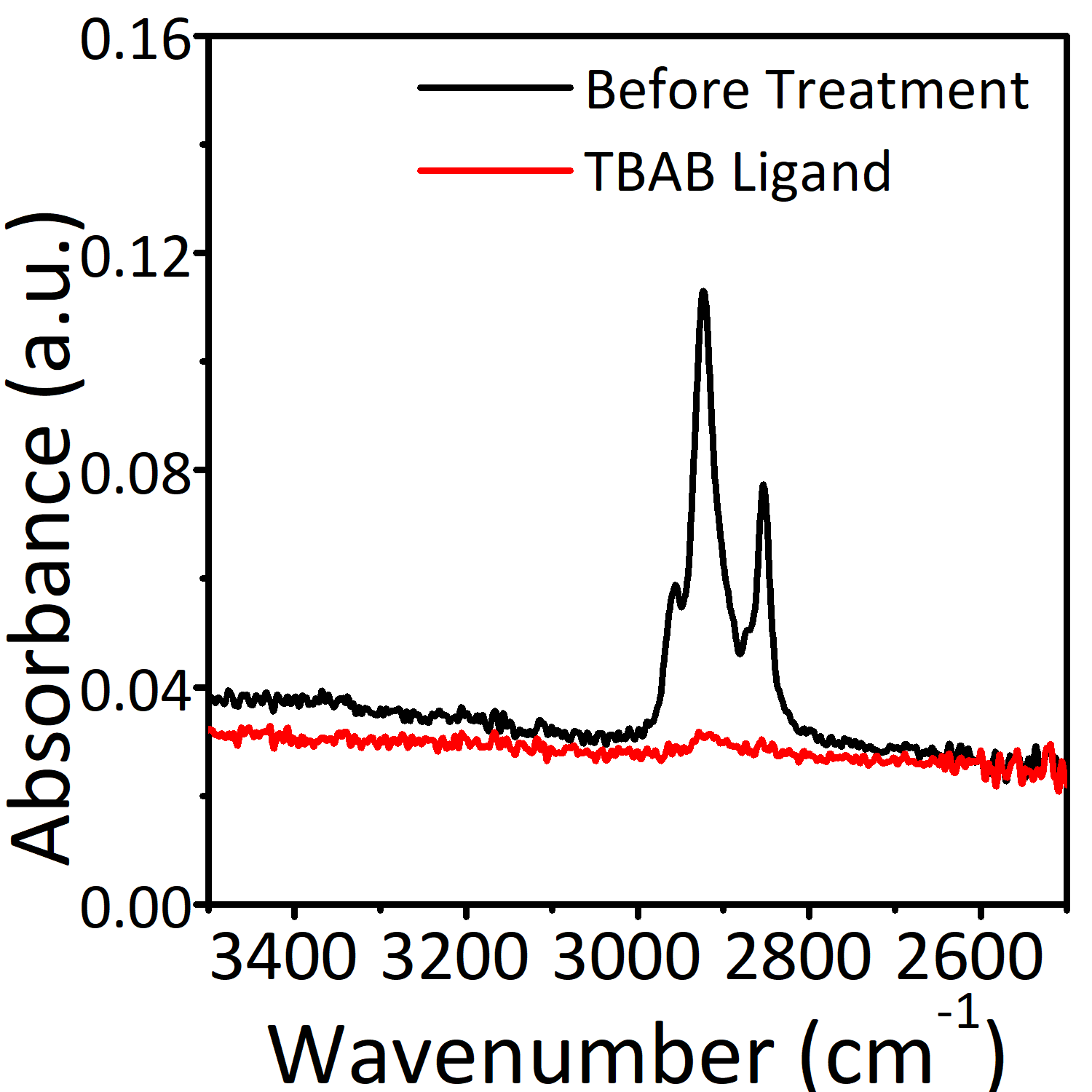


**Figure S15.** FT-IR spectra of BiSBr NWs a) before and b) after ligand exchange.


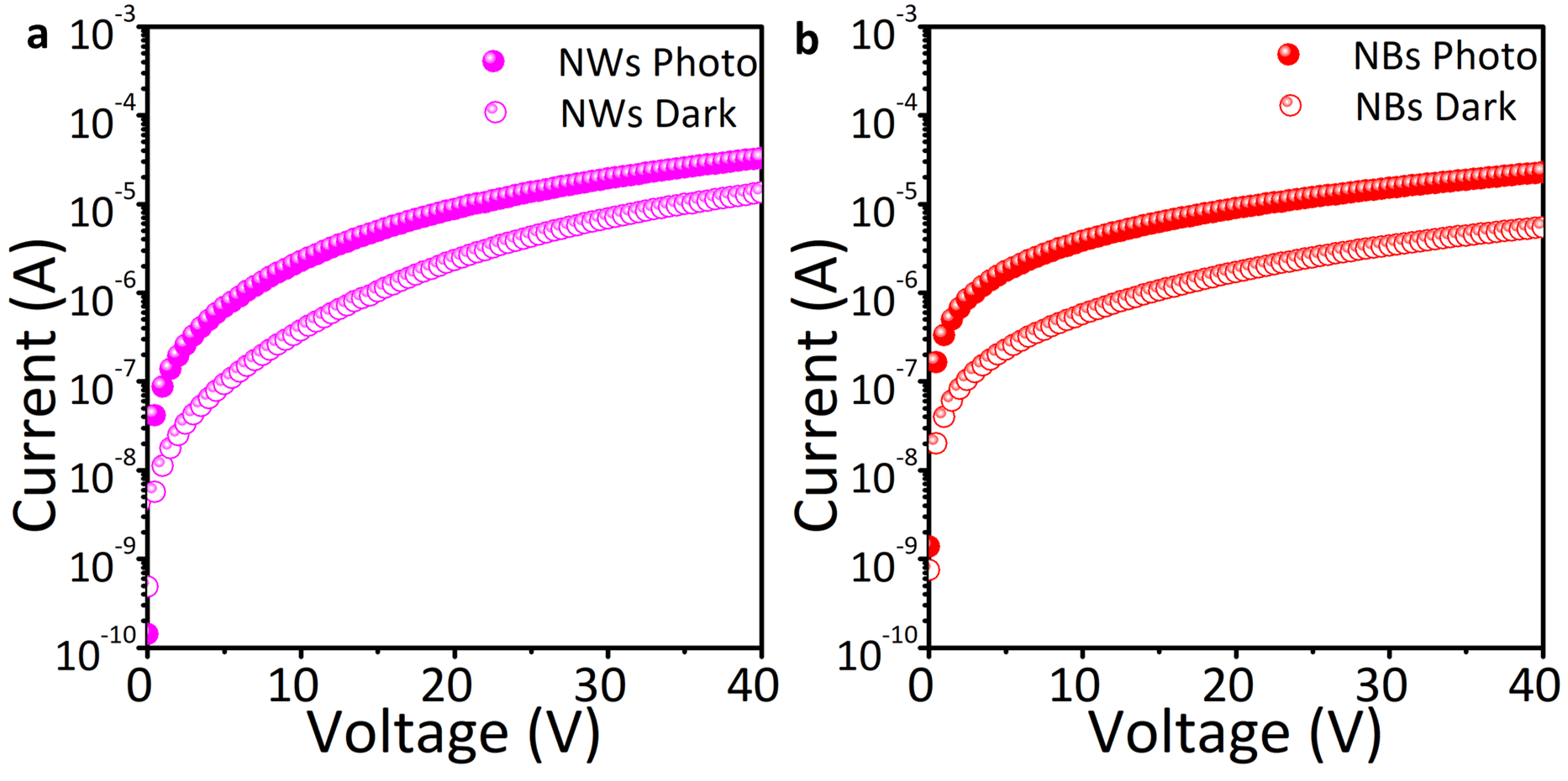


**Figure S16**. Current–voltage characteristics of a) BiSBr NWs and b) BiSBr NBs before ligand exchange under dark and illumination conditions. Measurements were performed under 532 nm laser illumination at a light intensity of 0.7 mW/cm².


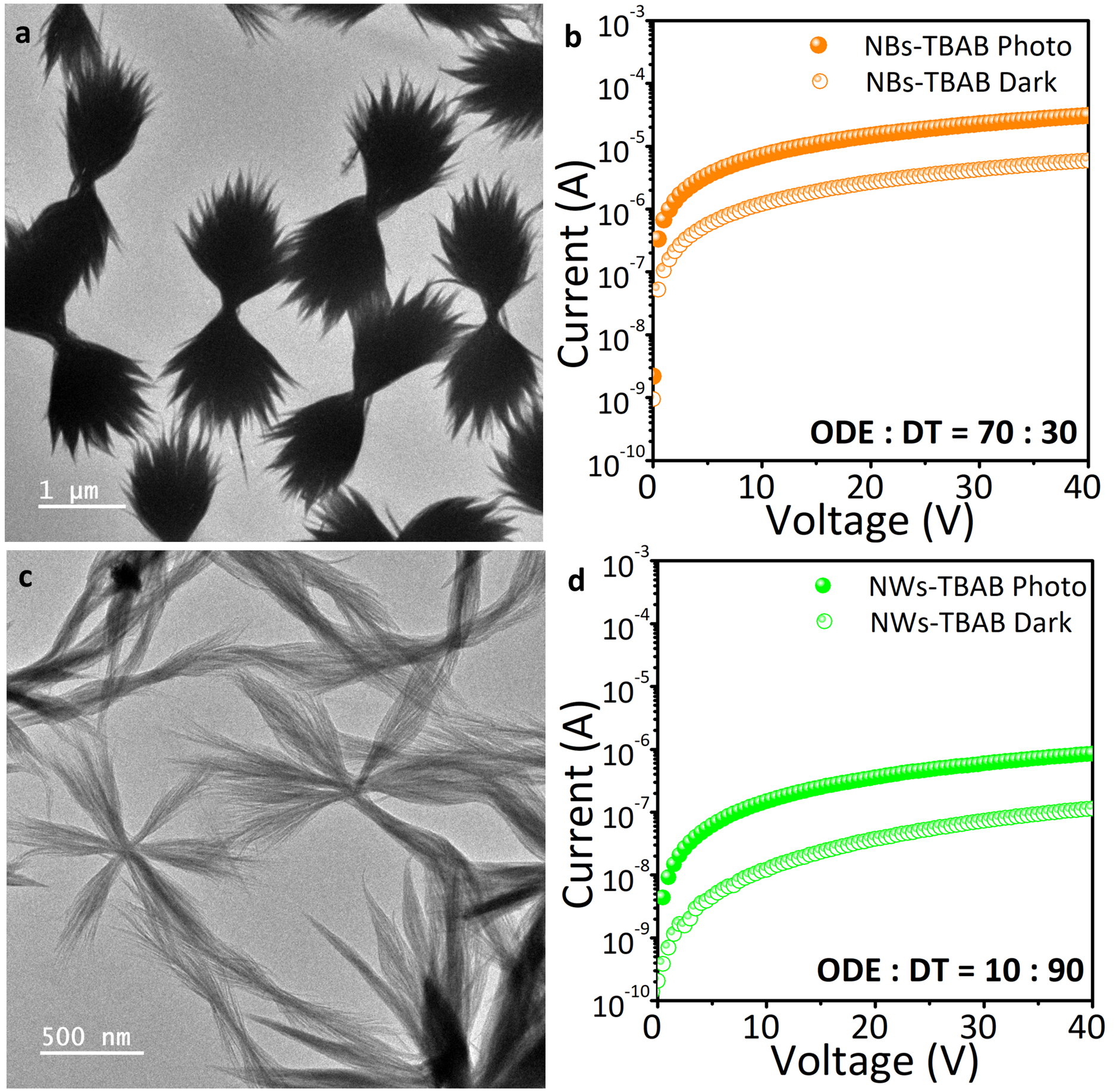


**Figure S17**. TEM images of BiSBr NBs used for photodetector fabrication and current–voltage characteristics under dark and illumination conditions for ligand-exchanged BiSBr NBs synthesized using ODE:DT volume ratios of a and b) 70:30 and c and d) 10:90. Measurements were conducted under 532 nm laser illumination at a light intensity of 0.7 mW/cm².

| Materials | Abs. λ | Fabrication method | Responsivity | Detectivity | Light Intensity | Substrate | Ref |
| --- | --- | --- | --- | --- | --- | --- | --- |
| CdSe NWs | Vis | CVD | 40 A/W | - | 520mW/cm^2^ | Rigid | [1] |
| Bi nanosheets | Vis | Liquid exfoliation | 2.95 uA/W | - | 100 mW/cm^2^ | Rigid | [2] |
| Bi nanosheets | Vis | Liquid exfoliation | 9.7 uA/W | - | 60 mW/cm^2^ | Flexible | [3] |
| BiOI | Vis | CVD | 2.6 × 10^-2^ A/W | 8.2 × 10^11^ | 0.283 mW/cm^2^ | Rigid | [4] |
| BiOBr | UV | CVD | 14.96 A/W | 5.7 × 10^10^ | 0.015 mW/cm^2^ | Rigid | [5] |
| BiOCl | UV | microwave | 8.0 A/W | - | 1 mW/cm^2^ | Flexible | [6] |
| BiSI | Vis | Solution processed | 62.1 A/W | 2.0 × 10^13^ | 0.0298 mW/cm^2^ | Rigid | [7] |
| BiSI | Vis | Wet chemical method | 64 nA/W | 1.27 × 10^8^ | 127 mW/cm^2^ | Rigid | [8] |
| BiSI | Vis | Wet chemical method | 8.7 nA/W | 6.3 × 10^6^ | 127 mW/cm^2^ | Flexible (no strain) | [8] |
| BiSeI | Vis | CVD | 5.88A/W | 1.3 × 10^3^ | 0.00002 mW | Rigid | [9] |
| BiSBr NWs, NBs | Vis | Solution processed | 36 A/W | 1.8 × 10^12^ | 0.029 mW/cm^2^ | Flexible | This work |
| BiSBr NWs, NBs | Vis | Solution processed | 158 A/W | 7.8 × 10^12^ | 0.0026 mW/cm^2^ | Flexible | This work |
| BiSBr NWs, NBs | Vis | Solution processed | 25.15 A/W | 7.1 × 10^11^ | 0.7 mW/cm^2^ | Flexible (no strain) | This work |

**Table S2**. Overview of photoconductors prepared using Bi-based materials in terms of different absorption spectra, fabrication methods, responsivity, detectivity, and substrate material.

REFERENCES

[1] E. Shalev; E. Oksenberg; K. Rechav; R. Popovitz-Biro; E. Joselevich, *ACS nano* **2017,** *11*, 213.

[2] H. Huang; X. Ren; Z. Li; H. Wang; Z. Huang; H. Qiao; P. Tang; J. Zhao; W. Liang; Y. Ge, *Nanotechnology* **2018,** *29*, 235201.

[3] B. Wang; Y. Zhou; Z. Huang; H. Qiao; C. Duan; X. Ren; Z. Wang; J. Zhong; X. Qi, *Mater. Today Nano.* **2021,** *14*, 100109.

[4] W. Zeng; J. Li; L. Feng; H. Pan; X. Zhang; H. Sun; Z. Liu, *Adv. Funct. Mater.* **2019,** *29*, 1900129.

[5] L. Chen; C. Yang; C. Yan, *J. Mater. Sci. Technol.* **2020,** *48*, 100.

[6] L. Kang; X. Yu; X. Zhao; Q. Ouyang; J. Di; M. Xu; D. Tian; W. Gan; C. C. Ang; S. Ning, *InfoMat* **2020,** *2*, 593.

[7] S. Farooq; T. Feeney; J. O. Mendes; V. Krishnamurthi; S. Walia; E. Della Gaspera; J. van Embden, *Adv. Funct. Mater.* **2021,** *31*, 2104788.

[8] K. Mistewicz; T. K. Das; B. Nowacki; A. Smalcerz; H. J. Kim; S. Hajra; M. Godzierz; O. Masiuchok, *Sci. Rep.* **2023,** *13*, 8800.

[9] Y. Li; S. Wang; J. Hong; N. Zhang; X. Wei; T. Zhu; Y. Zhang; Z. Xu; K. Liu; M. Jiang, *Small* **2023,** *19*, 2302623.
